# A Quantitative Framework for Flower Phenotyping in Cultivated Carnation (*Dianthus caryophyllus* L.)

**DOI:** 10.1371/journal.pone.0082165

**Published:** 2013-12-13

**Authors:** Borja Chacón, Roberto Ballester, Virginia Birlanga, Anne-Gaëlle Rolland-Lagan, José Manuel Pérez-Pérez

**Affiliations:** 1 Instituto de Bioingeniería, Universidad Miguel Hernández, Elche, Spain; 2 Department of Biology, University of Ottawa, Ottawa, Ontario, Canada; Instituto de Biología Molecular y Celular de Plantas, Spain

## Abstract

Most important breeding goals in ornamental crops are plant appearance and flower characteristics where selection is visually performed on direct offspring of crossings. We developed an image analysis toolbox for the acquisition of flower and petal images from cultivated carnation (*Dianthus caryophyllus* L.) that was validated by a detailed analysis of flower and petal size and shape in 78 commercial cultivars of *D. caryophyllus*, including 55 standard, 22 spray and 1 pot carnation cultivars. Correlation analyses allowed us to reduce the number of parameters accounting for the observed variation in flower and petal morphology. Convexity was used as a descriptor for the level of serration in flowers and petals. We used a landmark-based approach that allowed us to identify eight main principal components (PCs) accounting for most of the variance observed in petal shape. The effect and the strength of these PCs in standard and spray carnation cultivars are consistent with shared underlying mechanisms involved in the morphological diversification of petals in both subpopulations. Our results also indicate that neighbor-joining trees built with morphological data might infer certain phylogenetic relationships among carnation cultivars. Based on estimated broad-sense heritability values for some flower and petal features, different genetic determinants shall modulate the responses of flower and petal morphology to environmental cues in this species. We believe our image analysis toolbox could allow capturing flower variation in other species of high ornamental value.

## Introduction

*Dianthus* is a large genus with over 300 species (including carnations and pinks), a high incidence of polyploidy [1], and the fastest speciation rate reported to date in the flowering plants [2]. Most of the commercial carnation cultivars have been derived from *Dianthus caryophyllus* L., mostly by intra- but also by interspecific hybridizations with other *Dianthus* species [3]. Along with color pattern, petal shape and size is one of the most important targets in ornamental crop breeding. Breeding strategies to improve the quality of cultivated carnation need to consider a large number of characteristics from the cultivars selected, and are normally hindered by the polygenic nature of some of the traits selected [4].

A quantitative method for the evaluation of petal shape in *Primula sieboldii*, a popular garden perennial in Japan and north-east Asia, by use of elliptic Fourier descriptors and principal component analysis (EF-PCA) has been reported [5]. From the detailed analysis of 75 commercial cultivars and 9 wild populations, the authors reported four principal components (PCs) accounting for most of the variation found in petal shape in this species [6]. Quantitative changes that have occurred in petal shape during domestication, such as the prevalence of fan-shaped and large petals in cultivated *P. sieboldii*, were identified. Additionally, petal shape was found more stable and less influenced by the environment than petal size [6]. In another study using a single natural population of yellow monkeyflower (*Mimulus guttatus*), epistatic genetic interactions among quantitative trait loci (QTL) for flower size were identified, which were proposed to account for some of the evolutionary differences observed in flower features in *Mimulus* spp. [7], [8].

From recent studies in the *Arabidopsis thaliana* model plant, several genes were shown to regulate petal growth by affecting cell proliferation and/or cell expansion in an organ-specific manner [9]. Besides, only a few studies made use of the natural phenotypic variation to elucidate the genetic basis for quantitative variation in petal size and shape in this species [10], [11]. These analyses demonstrated that allelic differences at several loci can independently affect distinct petal size and shape features, indicating that the genetic regulation of each of these traits can occur in an independent manner [10], [11].

With the aim of creating an appropriate framework to study the genetic mechanisms and the environmental effects affecting flower morphology in ornamental plants, we set up a standardized procedure for the acquisition and analysis of flower and petal images. We present here our functional validation of this platform through a detailed analysis of flower and petal size and shape in 78 commercial varieties of cultivated carnation (*D. caryophyllus* L.).

## Materials and Methods

### Plant Material

The 55 standard carnation, 22 spray and 1 pot carnation cultivars used in this study are listed in Table S1. For each cultivar, about 40 rooted stem cuttings from several mother plants of the germplasm collection of Barberet & Blanc (http://www.barberet.es/) were transplanted in the 6^th^ June 2011 to substrate bags of pine bark, peat and perlite, and were grown in the same glasshouse under environmental conditions at 37°34′50″ N, 1°46′35″ W and 395 m altitude (Puerto Lumbreras, Murcia, Spain). Water, fertilizers and adequate phytosanitary treatments were periodically applied by skilled operators. In mature plants, primary stems were pinched according to standard procedures for homogeneous flower production. Every Monday between 7^th^ November 2011 and 19^th^ March 2012, 10 mature flowers at similar developmental stage from 10 different cultivars were harvested from several mother plants and stored in cardboard boxes at 4°C until shipment to the Universidad Miguel Hernández the next day. For some cultivars, two different samples (replicate 1 and replicate 2) were harvested. Since the flowers were randomly collected from vegetative-propagated (clonal) plants of the same cultivar, any difference between the flowers of the same cultivar in a given sample might mostly be attributed to the effect of the environment (Figure S1).

### Sample Collection

**Figure 1:**
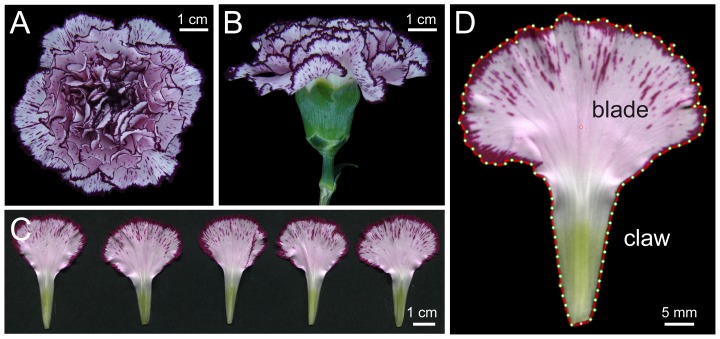
Morphological characterization of *D. caryophyllus* flowers. Examples of flowers (A, top view, B, side view) and petals (C, D) from *D. caryophyllus* cv. ‘Falicon’. In C, five representative petals of the outer whorl of the corolla from a given flower are shown. In D, an image output of a given petal as obtained from LeafAnalyser is shown; red line delimits the petal margin, white dots indicate petal centroids and petal base tips, respectively, and green dots are evenly distributed landmarks (n = 96) along the petal margin. Flower and petal photographs were obtained as described in Materials and methods.

After receiving the samples, cut flowers were kept watered in a growth chamber at 25±2°C, 60% RH and 14∶10 h (light:dark) photoperiod with an average photosynthetic photon flux density of 80 µmol m^−2^ s^−1^. From each sample, 4 flowers at the same developmental stage (when the angle between the calix and the outer petals was 85° to 115°) were chosen at random from the 10 flowers received. For each flower, top, side and bottom view pictures were taken using a Sony Cyber-shot DSC-H3 camera (Sony Corporation, Tokyo, Japan) at a resolution of 3264×2448 pixels, and saved as an RGB color image in jpeg format (Figure 1A–B). Five out of the seven petals taken at random from the outer whorl of the corolla in each flower and mounted on a black cardboard (Figure S1). Petals were flattened for 2 h on a wooden press before being scanned, at a resolution of 800 dpi and 24-bit color, on an Epson Perfection V330 Photo scanner (Seiko Epson Corporation, Nagano, Japan) and saved as an RGB color image in jpeg format (Figure 1C). After scanning, the flattened petals on the cardboard were kept for one week in a wooden press, and later stored in transparent plastic sleeves.

### Image Analysis

For the morphometric analysis of flower images, we used ImageJ 1.40 g (http://rsb.info.nih.gov/ij/). We have written an ImageJ macro to batch process the images and to extract quantitative information of corolla features. As the red-channel gave the clearest contrast between the flowers and the background, the RGB images were converted into binary images by applying a threshold method to the red channel. Measurements were taken for flower area (FA) and perimeter (FP), major (FEM) and minor (FEN) chord lengths of the best fitting ellipse containing the flower, as well as area (FHA) and perimeter (FHP) of the convex hull (i.e. the smallest convex set that contained the flower). Some shape factors were also measured, such as the aspect ratio (FAR = major chord/minor chord), flower solidity (FS = flower area/convex hull area) and flower convexity (FC = convex hull perimeter/flower perimeter).

For the morphometric analysis of petal scans, we used MATLAB Version 7.13 (R2011b; http://www.mathworks.es/products/matlab/) to write algorithms and a graphical user interface for automated analysis of petal characteristics. Those algorithms are part of more general software for carnation shape and color pattern analysis and will be published elsewhere (Rolland-Lagan et al., in preparation). In brief, algorithms were developed to automatically process the petal database containing images from each cultivar. For each image, the algorithm extracted the petal outline as well as relevant morphometric parameters (Table S2). The outline data were saved as.mat files and leaf parameters were recorded in an Excel file for their statistical analysis.

In order to further analyze characteristics of petal shapes, we used LeafAnalyser (http://leafanalyser.openillusionist.org.uk/doku.php) to define the contour of each petal based on 96 spatial landmarks and by taking as a reference (y = 0) the basal tip of the claw (Figure 1D). The x,y spatial coordinates were used in a principal component (PC) analysis as described in [12] to determine the morphological models accounting for the variation found in our petal sample population. Average values for each PC were plotted using the analysis options from LeafAnalyser.

### Heat Map Representation

Morphometric data obtained from the analysis of carnation flowers and petals were processed using the heatmap.plus package of R (http://www.r-project.org/). Neighbor-joining distance matrixes between cultivars (rows) and between morphological parameters (columns) were calculated to build the dendrograms.

### Statistical Analysis

Descriptive statistics (mean, standard deviation [SD], maximum and minimum) and linear correlations were calculated by using the StatGraphics Centurion XV software (http://www.statgraphics.com/). The differences between the data groups were analyzed by t test (*P*<0.05) when only two groups were compared. One-sample Kolmogorov-Smirnov tests [13] were performed to analyze the goodness-of-fit between the distribution of the data and a theoretical distribution. To compare the data for a given variable among the different cultivars studied, we performed multiple testing analyses with the ANOVA F-test or the Fisher’s LSD (Least Significant Differences) methods [14]. Non parametric tests were used when necessary. In that case median was used instead of mean. The differences between the data groups were analyzed by Mann-Whitney U test (*P*<0.05) when only two groups were compared. In the other cases, data were subjected to Kruskal-Wallis test (*P*<0.05). Correlations were studied using Pearson product-moment correlation coefficient (Pearson’s r).

### Trait Heritability and Norm of Reaction Estimates

Broad-sense heritability (*H^2^*) for a given character is defined as the proportion of the phenotypic variation (V_F_) that might be attributed to genetic factors (V_G_). The simplest approach to estimating *H^2^* in clonally propagated species, such as carnation, is to perform one-way ANOVA test as described previously [15]. According to our experimental design, for N independent genotypes in each population (55 standard carnation cultivars and 23 spray and pot carnation cultivars, respectively), the within-genotype mean square value (MS_W_) is an estimate of the environmental variance (V_E_) while the among-genotype mean square value (MS_A_) represents V_E_+n×V_G_, hence 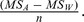 As the number of flower and petal samples for a given genotype depends on the number of replicates collected, we estimated n as the average number of samples (either flowers or petals) in each population. To estimate *H^2^* in the whole population of 78 carnation cultivars, we transformed the data to the standard normal distribution, *N* (0, 1). Genetic correlations (*r_G_*) were estimated as 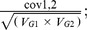 where cov1,2 is the covariance of trait values and V_G1_ and V_G2_ are the among-genotype variance components for those traits. The coefficient of genetic variation (CV_G_) was estimated for each trait as: 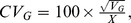 where V_G_ is the among-genotype variance component and X is the trait mean of the genotypes [16]. To estimate the reaction norms for cultivars with two replicates collected in different seasons, we transformed the data in each subpopulation to the standard normal distribution, *N* (0, 1). Next, the data was represented graphically and linear regression models were built using the least squares method.

## Results

### Quantitative Phenotyping of Flower Morphology in Cultivated Carnation

To provide a conceptual framework for the quantitative description of flower morphology in cultivated carnation, we collected flower and petal samples from 55 standard carnation, 22 spray carnation and 1 pot carnation cultivars (Table S1; see Materials and methods). We measured 28 parameters in flower images at different views (top, t; side, s; bottom, b) and 9 parameters in excised petals from the outer whorl (Table S2). Correlation analyses (Table S3A) allowed us to reduce the number of parameters accounting for the variation in flower morphology to only four: flower area (FA), flower aspect ratio (FAR), flower solidity (FS) and flower convexity (FC). Hereafter only data from top view flower images will be shown. Variation in the recorded petal morphology in the studied populations could be best described with just three variables: petal area (PA), petal solidity (PS) and petal convexity (PC). Petal perimeter (PP) was found highly significant and negatively correlated with PC (Table S4A). Interestingly, only in petals but not in flowers, the area (PA) was highly significant and negatively correlated with their aspect ratio (PAR) (Table S4A).

### Flower Size and Shape Distribution in Cultivated Carnation

**Figure 2:**
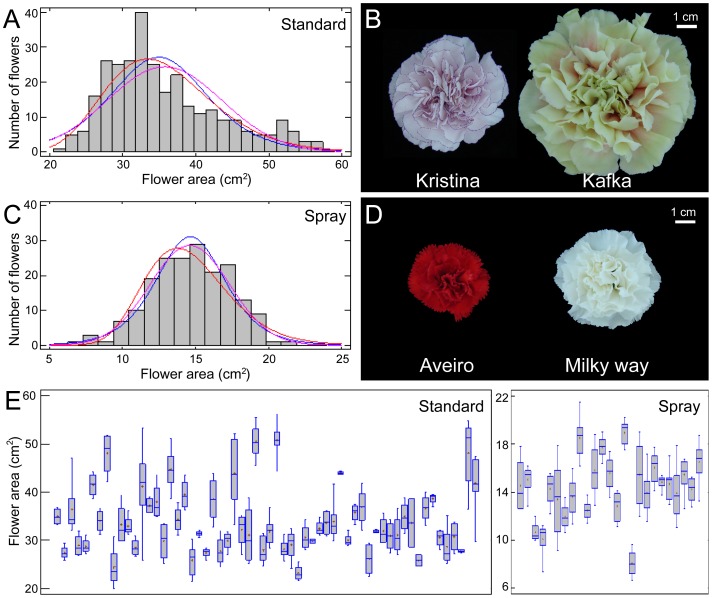
Flower size distribution among carnation cultivars. (A, C) Histograms for the studied flower area dataset with overlay of theoretical distributions (blue, normal distribution; red, log-normal distribution; pink, logistic distribution) in standard (A) and spray (C) cultivars. (B, D) Representative photographs of flowers with extreme values for area in standard (B) and spray (D) cultivars. (E) Box-plots of flower area for the studied cultivars. Flower photographs were obtained and analyzed as described in Materials and methods.

In standard carnation, FA data fit the log-normal distribution function (*P*<0.05). We found a two-fold range in average values for flower area between the extremes in this population (Figure 2A), represented by the cultivars Kristina (24.20±1.63 cm^2^) and Kafka (51.49±3.37 cm^2^) (Figure 2B). In spray carnation, the FA values fit the Gaussian distribution function (Figure 2C; *P*<0.05), and ranged between 10.12±1.27 cm^2^ (Aveiro) and 18.94±1.02 cm^2^ (Milky Way) (Figure 2D). Interestingly, flower size in both populations partially overlapped (Figure 2E and Table S3B) hence the flowers in Kristina (standard) and Milky Way (spray) cultivars displayed similar sizes. In the studied populations, average FP values showed a six-fold range (Figure S2A, S2C) between Mondriaan and Roble cultivars (Figure S2B, S2D).

**Figure 3:**
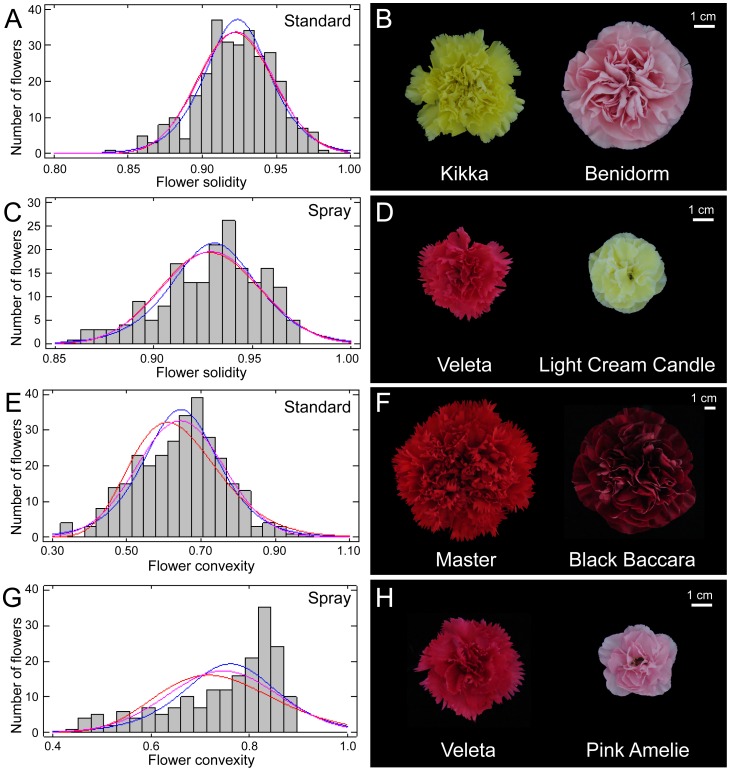
Flower evenness among carnation cultivars. (A, C) Histograms for the flower solidity dataset with overlay of theoretical distributions (blue, normal distribution; red, log-normal distribution; pink, logistic distribution) in standard (A) and spray (C) cultivars. (B, D) Representative photographs of flowers with extreme values for solidity in standard (B) and spray (D) cultivars. (E, G) Histograms for the flower convexity dataset with overlay of theoretical distributions (blue, normal distribution; red, log-normal distribution; pink, logistic distribution) in standard (E) and spray (G) cultivars. (F, H) Representative photographs of flowers with extreme values for convexity in standard (F) and spray (H) cultivars. Flower photographs were obtained and analyzed as described in Materials and methods.

Several parameters accounted for flower shape. Both in the standard carnation and in the spray carnation populations, FAR values ranged between 1.01 and 1.20 (Figure S2E, S2G). Since FAR relates to the proportional relationship between the longest and the shortest axis of the flower, higher FAR values (Rita, standard; Amelie, spray) account for elliptical shapes of the flowers while values approaching one (Duque, standard; Pink Amelie; spray) indicate their circular shape (Figure S2F, S2H). Besides, FS associates the area of the flower with the area of its convex hull, such that homogeneous flowers will display the highest value (FS = 1). In both populations, FS values fit the Gaussian distribution function (*P*<0.05), and ranged from 0.86 (Kikka, standard; Veleta, spray) to 0.98 (Benidorm, standard; Light Cream Candle, spray) (Figure 3A–D). Another shape parameter is FC, which associates the perimeter and the convex hull perimeter of the flower. In flowers with entire petals, FC values will reach one, and these values will decrease as flowers become more serrated. Whereas in standard carnation, FC data fit the normal distribution (Figure 3E; *P*<0.05) with average values ranging from 0.448±0.031 (Master) to 0.939±0.024 (Black Baccara) (Figure 3F), in spray carnation, FC data were unevenly distributed (Figure 3G), with the extremes represented by Veleta (0.490±0.027) and Pink Amelie (0.856±0.021) (Figure 3H).

### Petal Size and Shape Distribution in Cultivated Carnation

**Figure 4:**
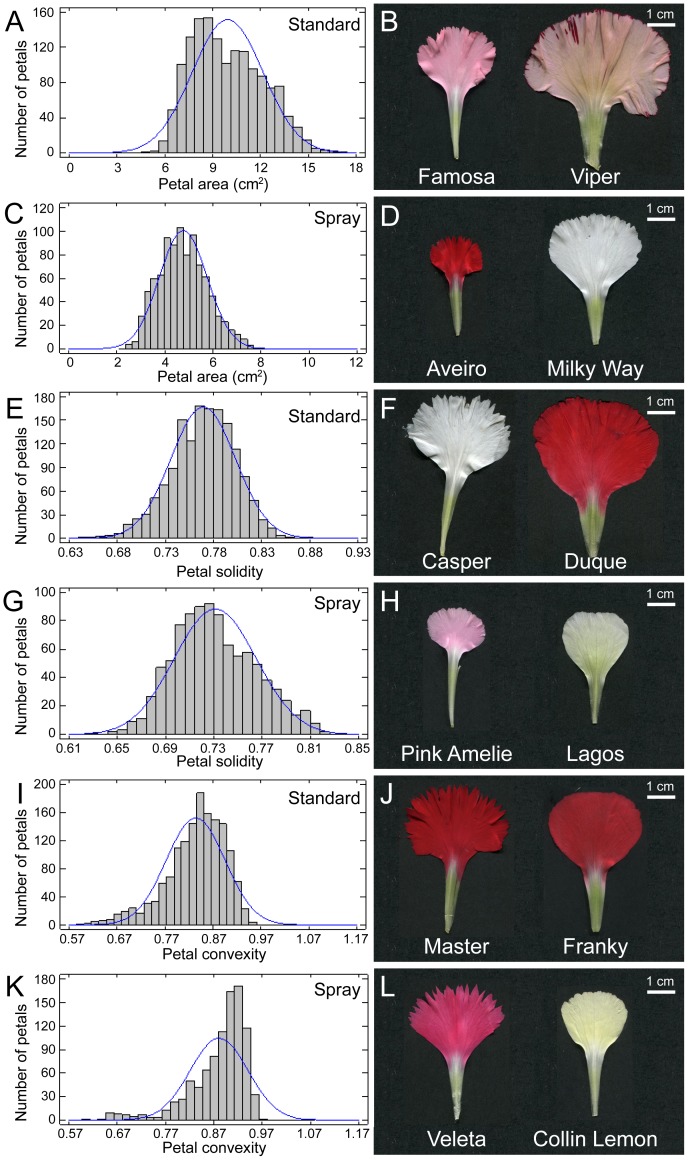
Petal morphometry among carnation cultivars. (A, C) Histograms for the petal area dataset with overlay of normal theoretical distribution in standard (A) and spray (C) cultivars. (B, D) Representative photographs of petals with extreme values for area in standard (B) and spray (D) cultivars. (E, G) Histograms for the petal solidity dataset with overlay of normal theoretical distribution in standard (E) and spray (G) cultivars. (F, H) Representative photographs of petals with extreme values for solidity in standard (F) and spray (H) cultivars. (I, K) Histograms for the petal convexity dataset with overlay of normal theoretical distribution in standard (I) and spray (K) cultivars. (J, L) Representative photographs of petals with extreme values for area in standard (J) and spray (L) cultivars. Petal images were obtained and analyzed as described in Materials and methods.

In standard carnation, average PA ranged from 6.61±0.40 cm^2^ in Famosa to 11.95±1.64 cm^2^ in Viper (Figure 4A–B). Mondriaan (pot carnation) and Aveiro (spray carnation) were the cultivars with the smallest petal area values (<3 cm^2^) (Figure 4C–D). As for the flower area (see above), PA values of the largest spray carnation cultivar (Milky Way; 6.43±0.57 cm^2^) overlapped with those of the smallest standard carnation cultivar (Famosa) (Table S4B). Although we also measured PP (Figure S3A–D), this parameter was not considered a useful descriptor for petal shape. PAR values fit the normal distribution function in standard carnation and spray carnation cultivars (Figure S3E, S3G; *P*<0.05). Elongated petals (PAR>1.5) were more frequently found in spray carnation than in standard carnation (Figure S3E, S3G), with the highest values of 1.81 in Falicon (standard) and of 2.22 in Collin (spray) (Figure S3F, S3H). On the other hand, the lowest PAR values were represented by petals of Viper (1.04; standard) and Lagos (1.29; spray) (Figure S3F, S3H).

Values of PS close to 1 are characteristic of petals with large and entire blades and short and wide claws, such as those in Duque (0.86; standard) or Lagos (0.82; spray), while petals in Casper (standard) and Pink Amelie (spray) displayed an average PS value below 0.65 (Figure 4E–H). On the other hand, PC is a good descriptor for the serration of the petal margin: PC values approaching 1 are characteristic of petals with entire margins, while the lowest PC values correspond to serrated petals. PC values did not fit the Gaussian distribution function in the studied populations (Figure 4I, 4K), and most cultivars displayed entire petals, such as those in Franky (standard) and Collin Lemon (spray) (Figure 4J, 4L). The highest serration values were found in petals from Casper and Master in standard carnation, and White Ashley and Veleta in spray carnation (Figure 4J, 4L and Table S4B).

### Correlations between Morphometric Parameters in Flowers and Petals

**Figure 5:**
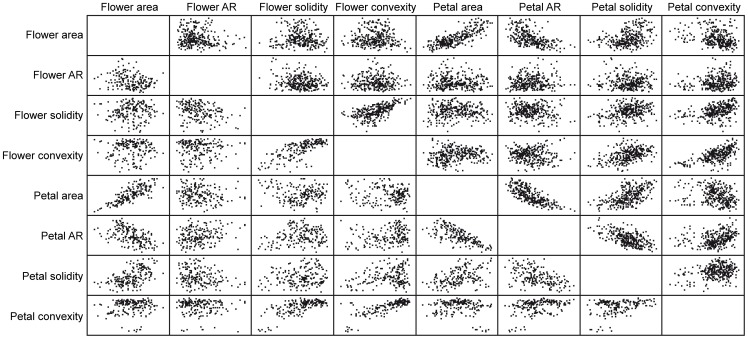
Scatter plots of flower and petal parameters. The blank diagonal separates the graphs for the standard cultivar dataset (right upper area of the rectangle) from those of the spray cultivar dataset (left lower area of the rectangle).

Flower morphology is highly dependent on several parameters associated with the corolla structure, such as the size and the shape of individual petals, their petal number or the phyllotactic arrangement of the petals in the stem. Also, calix structure can influence flower morphology to some extent. We wondered whether the flower and petal parameters measured in our study were statistically correlated (Table S5). In standard carnation cultivars (Figure 5, right upper area of the rectangle), we found highly significant and positive correlations for FA and PA (r = 0.794; *P*<0.005) and between FC and PC (r = 0.595; *P*<0.005). Conversely, the other parameters estimating size and shape in flowers and petals were only slightly correlated and were not considered biologically relevant. Similar results were found for the correlations between areas (r = 0.789; *P*<0.005) and convexities (r = 0.790; *P*<0.005) in spray carnation cultivars (Figure 5, left down area of the rectangle, and Table S5). Additionally, the correlations found between FS and PC were statistically significant only in spray carnation cultivars (r = 0.693; *P*<0.005). These results suggest that, in cultivated carnation, the shape and the size of petals from the outer whorl, as those measured in our analysis, are good descriptors of the overall differences in flower morphology among carnation cultivars.

### Integration and Visualization of Morphological Data

**Figure 6:**
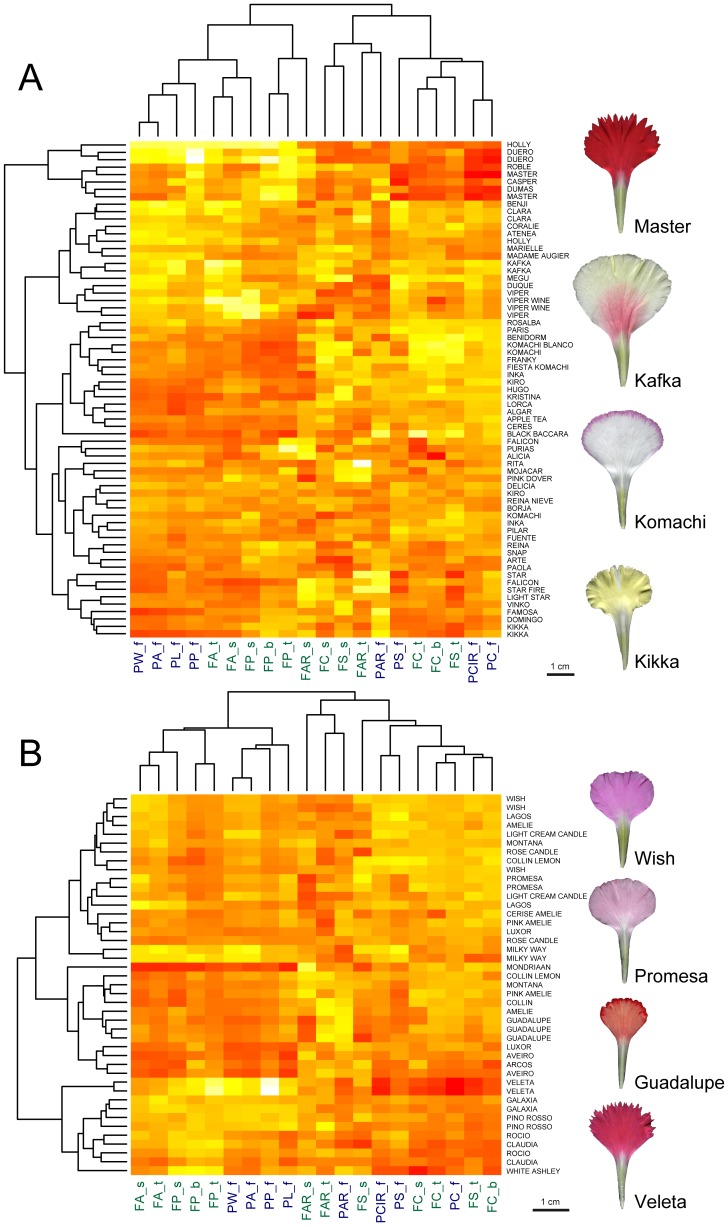
Morphological characteristics in cultivated carnation. Heat map representation of (A) standard and (B) spray carnation cultivars. The morphological parameters analyzed from flower images (blue; top, t; bottom, b; side, s) and petals (green) are shown in the row below the heat map (see Materials and methods). The color code in the histogram goes from red (lowest values) to white (highest values).

As a method to easily evaluate the similarity between the cultivars studied, we selected 20 parameters (12 from flowers and 8 from petals) to build heat map representations with our data (Figure 6). Several parameters defining size, such as area, length or width, either in flowers or in petals, were grouped in the same node of the upper dendrogram, while the parameters that determined the shape of these organs clustered in a different node.

In 7 of the 12 standard carnation cultivars with two replicates in the experiment, both replicates were pooled in the same tree node of the cultivar dendrogram shown in the left (Figure 6A). In addition, cultivars with a common origin, such as Komachi, Komachi Blanco and Fiesta Komachi, Star and Star Fire, or Viper and Viper Wine, also appear clustered together in the cultivar dendrogram. These results indicate that the cultivar dendrogram built with morphological data might provide some information about the phylogenetic relationships of the studied population. Standard cultivars with large and entire flowers, such as Kafka, were found distributed in the central region of the cultivar dendrogram, while cultivars with small flowers were found in the lower side of the dendrogram. Serrated cultivars, such as Master and Roble are clustered together in the upper side of the dendrogram.

In spray carnation, samples from 10 of the 18 cultivars with two replicates laid close together on the dendrogram (Figure 6B). However, the cultivar dendrogran does not capture all the morphological differences among spray carnation cultivars, likely because of the small range of variation found in spray carnation and/or the low number of cultivars tested in comparison with standard carnation. Note that Veleta is clustered apart from all the other spray carnation cultivars (Figure 6B).

### Quantitative Analysis of Petal Shape and Size in Carnation

**Figure 7:**
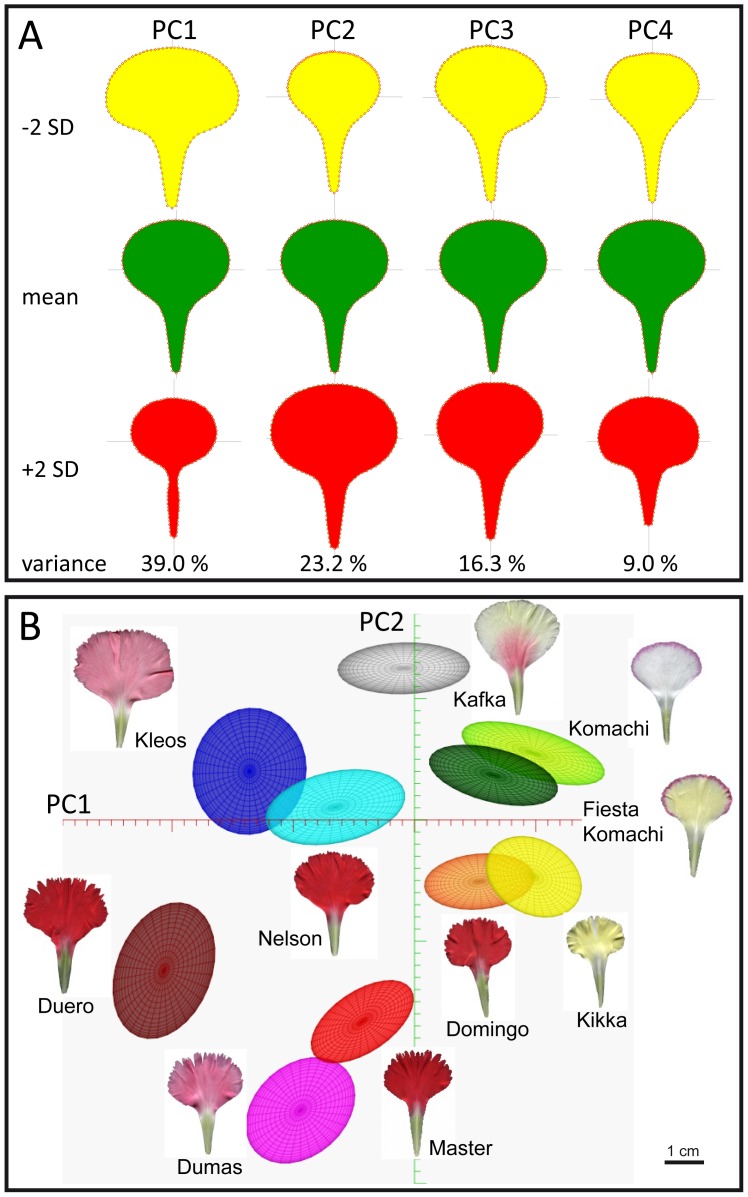
Principal component (PC) analysis of petal morphometry in standard carnation cultivars. (A) Petal shape by variation along the first four PCs of the carnation petal shape model, PC1 to PC4. The mean petal and petal shapes corresponding to PC values deviating by ±2 SD are shown. (B) Two-dimensional PC map generated by standard deviations from the mean petal along PC1 and PC2. The ellipses represent 1 SD from the mean petal for each one of the nodes (cultivars) shown.

In petals of standard carnation, four principal components (PCs) accounted for 87.5% of the morphological variation observed (Figure 7A; see Materials and methods). PC1 explained 39.0% of the total variance and was dependent on blade size and on the distal width of the claw. PC2, which accounted for 23.2% of the variance, was mainly dependent on blade area. 16.3% of the observed variance was explained by PC3, which seems to account for petal asymmetry along the medio-lateral axis, as the angles between the claw and the blade might differ within a single petal. Although PC4 captured only 9.0% of the observed variation, it seems to determine remarkable petal shape attributes, hence extreme values displayed obovate (−2 SD) and reniform (+2 SD) shape of the blade. PC1, PC2 and PC4 captured most of the genetic variation in shape and size in carnation petals, allowing their representation in a three-dimensional space. As expected, similar positions on this space were occupied by petals from the same cultivar or from morphologically similar cultivars (Figure S4). Some cultivars representing the variation spectra found in petal size and shape are shown in Figure 7B, which illustrates both non-overlapping and partially overlapping patterns of space occupancy, thus Dumas, Kafka and Kikka displayed contrasting petal phenotypes and Komachi and Fiesta Komachi showed petals differing mostly in their color patterns. Interestingly, our PC analysis allowed us to sort out petals based on the serration index, with Duero and Komachi as the extremes of the distribution observed for this parameter (Figure 7B).

**Figure 8:**
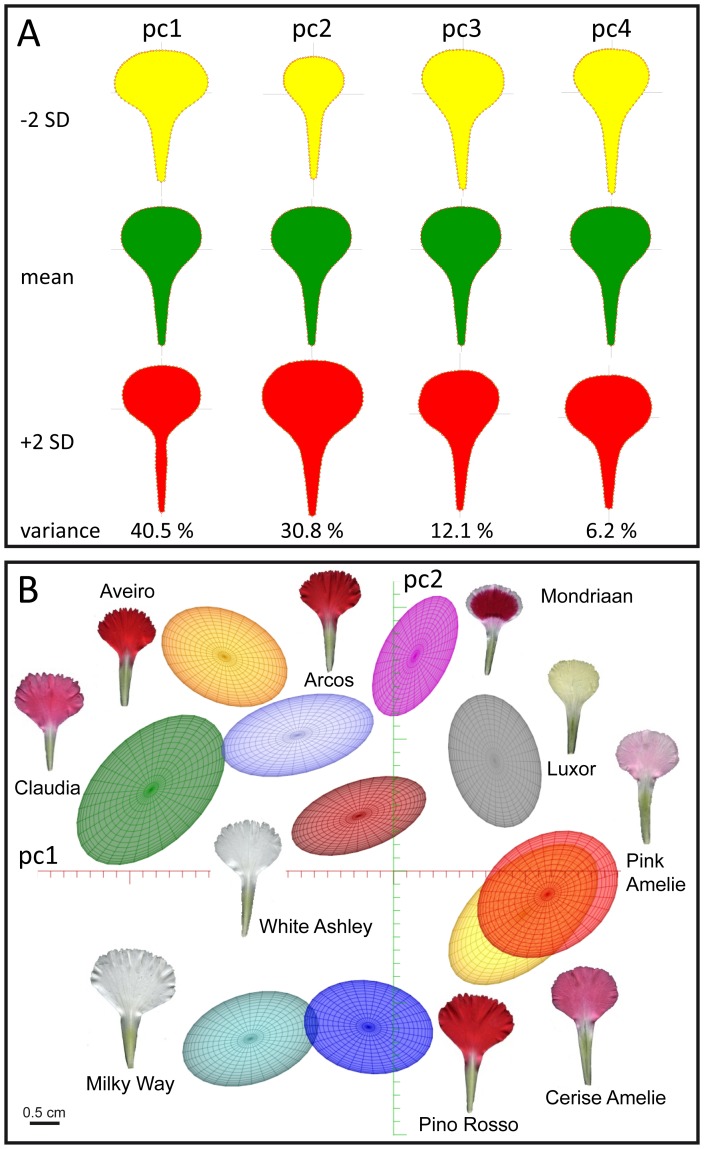
Principal component (PC) analysis of of petal morphometry in spray carnation cultivars. (A) Petal shape by variation along the first four PCs of the carnation petal shape model, pc1 to pc4. The mean petal and petal shapes corresponding to pc values deviating by ±2 SD are shown. (B) Two-dimensional pc map generated by standard deviations from the mean petal along pc1 and pc2. The ellipses represent 1 SD from the mean petal for each one of the nodes (cultivars) shown.

Similarly, we determined that 89.6% of the variation found among spray carnation petals was represented by four PCs, named pc1 to pc4 hereafter (Figure 8A; see Materials and methods). Interestingly, the overall petal features explained by pc1, pc2 and pc3 were very much similar in spray and standard carnation cultivars (see above). Despite pc1 and pc2 were mostly influenced by the morphology of the claw in spray carnation petals, PC1 and PC2 were strongly dependent on blade size in standard cultivars. pc4 accounted for 6.2% of the variation found in spray carnation petals, and was defined by the morphology features of the region located between the blade and the claw. Ten cultivars covering the morphological variation found in spray carnation petals are represented in Figure 8B. As shown above for standard cultivars, overlapping distribution of petal morphologies in spray carnation cultivars are indicative of a common origin, such in those of Cerise Amelie and Pink Amelie. In contrast, serration index was not accurately represented in our PC analysis.

### Broad-sense Heritability and Norm of Reaction of Flower and Petal Morphology in Carnation

According to our experimental design (Figure S1), broad-sense heritability (*H^2^*) could be estimated from a one-way ANOVA test only on those parameters that best described size and serration, such as the area and the convexity, respectively (see Materials and methods). *H^2^*, CV_G_ and r_G_ parameters for these flower and petal traits were estimated in standard carnation or in spray carnation independently, and also in the entire population of 78 cultivars (Table 1; see Materials and methods). In all cases, FC displayed higher *H^2^* values than FA. The genetic correlation indexes found between FA and FC were very low, suggesting that independent genetic factors contributed to the differences found for these traits in the studied population. In petals, *H^2^* values were slightly higher in the spray carnation than in standard carnation (Table 1). Both in flowers and in petals, traits measuring serration, FC and PC, displayed higher *H^2^* values than traits measuring size, such as FA and PA. Interestingly, we found negative genetic correlations between PA and PC (Table 1). These results are in agreement with the statistical correlation found between petal size and serration index, hence large petals displayed highly dissected margins and low convexity values (see above). Taken together, our results are indicative of shared genetic factors contributing to the differences observed in petal size and serration among carnation cultivars.

**Table 1:** Estimates of broad-sense heritability (*H^2^*) for some parameters.

	Area		Convexity		
Flower morphology^a^	*H^2^*	CV_G_	*H^2^*	CV_G_	r_G_
Standard carnation, n = 5	0.745	18.755	0.831	17.115	0.022
Spray carnation, n = 8	0.661	15.537	0.806	13.991	0.005
Entire population, n = 6	0.703	nd	0.816	nd	0.016
**Petal morphology**	***H^2^***	**CV_G_**	***H^2^***	**CV_G_**	**r_G_**
Standard carnation, n = 24	0.626	18.288	0.732	6.569	−0.386
Spray carnation, n = 37	0.658	17.779	0.856	6.675	−0.182
Entire population, n = 28	0.634	nd	0.778	nd	−0.307

^a^ n indicates the average number of samples in each population (see Materials and methods). nd: not determined.

**Figure 9:**
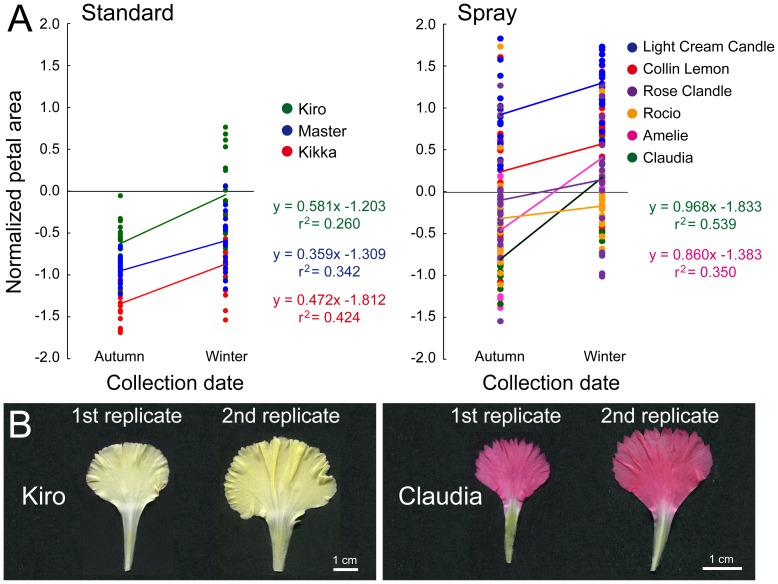
Norms of reaction for petal size in carnation cultivars collected at different environments. (A) Normalized petal area in standard and spray carnation cultivars with samples collected in autumn and in winter. (B) Representative petal images of carnation cultivars with extreme variations in their norm of reaction for petal area. Kiro, standard carnation; Claudia, spray carnation.

We found positive and significant correlations between FA and collection date in standard carnation cultivars (r = 0.721; *P*<0.005) and spray carnation cultivars (r = 0.368; *P*<0.005), while FC data and collection date were not statistically correlated (Figure S5). In petals, a significant correlation was found between PA and collection date only in standard carnation cultivars (r = 0.491; *P*<0.005) (Figure S5). These results suggested that seasonal differences might affect flower and petal size along the experiment, mainly in standard carnation cultivars. For the 30 cultivars with two replicates, only 3 standard carnation cultivars and 6 spray carnation cultivars contain the first replicate collected in autumn and the second one in winter (Figure 9A). Therefore we could estimate the norm of reaction for petal size in each of these genotypes in response to the autumn and winter environments (see Materials and methods). In all the studied cases, samples collected in winter display larger petals than those collected during autumn but the range of phenotypes for a single genotype varied among cultivars; Kiro (standard) and Claudia and Amelie (spray) cultivars displayed the largest variation in petal area (Figure 9A–B).

**Figure 10:**
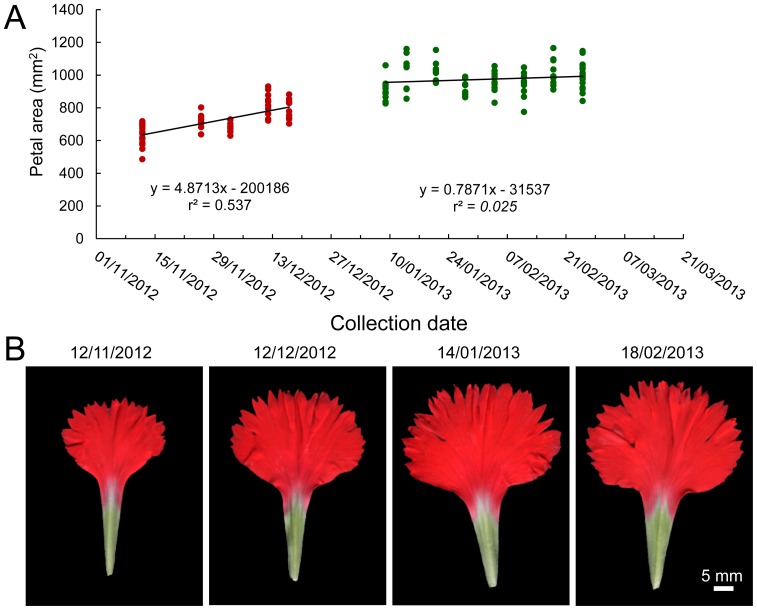
Environmental-dependency of petal growth in *D. caryophyllus* cv. ‘Master’. (A) Data from petal area of the standard carnation cultivar Master are represented over collection date. Linear regressions performed on data collected during different seasons (autumn, red; winter, green) are also shown. (B) Representative images of petals from the Master cultivar samples obtained at regular one-month intervals.

To confirm that the effects on the measured petal parameters were indeed caused by seasonal differences during the experiment, we next collected 13 samples from the same mother plants of the Master reference carnation cultivar between 12^th^ November 2012 and 25^th^ February 2013. PA increased during autumn and it was found statistically correlated with collection date until the end of December (Figure 10). In those samples collected from January onwards (winter season), PA did not increased significantly (Figure 10). These results confirmed that during autumn, the flowers from Master not only were smaller than those collected during winter but the final size of their petals increased constantly along the experiment until they reached a maximum size during winter.

## Discussion

Most important breeding goals in ornamental crops are plant appearance and flower characteristics where selection is visually performed on direct offspring of crossings. Carnation is, after rose, the most important species on the worldwide market of cut flowers, with a yearly sales volume of almost 200 million plants [3]. We present here a systematic approach for acquiring and analyzing images taken from flowers and petals of cultivated carnation. In addition, we performed a detailed analysis of flower and petal morphometry in 78 commercial cultivars of carnation, including 55 standard, 22 spray and 1 pot carnation.

Among the parameters analyzed in flowers, the area from the top view is the best estimate for flower size. Flower size distribution varies extensively between standard and spray carnation, with a 40-cm^2^ range represented by Aveiro and Kafka, and a minimal overlap between the two supbopulations. Flower size is an important breeding target, and in the case of cultivated carnation, consumers from different regions show size-specific preferences (E.A. Cano, personal communication). Other features of breeding interest in flowers are serration and asymmetry. Convexity is a measure of object roughness, and we used it as a descriptor for the degree of flower serration. A smooth (entire) flower has a convexity value close to 1 (Pink Amelie and Black Baccara), while serrated flowers have convexity values near 0.5 (Veleta and Master). Interestingly, in spray carnation, most of the studied cultivars displayed entire flowers, which might indicate positive selection for this trait during breeding. Two other parameters measured accounted for flower asymmetry: aspect ratio and solidity. A perfect circular flower has an aspect ratio of 1, and elliptical flowers will display values higher than 1. In both subpopulations, flower aspect ratio was centered on the 1.05 value and only a few cultivars displayed slightly elliptical flowers (Rita), a result that is indicative of the consumer preference for rounded flowers of radial symmetry; alternatively, it might be difficult to obtain carnation flowers of bilateral symmetry by breeding current carnation germplasm. We found that flower solidity is a complex descriptor for both serration and flower asymmetry. For example, flowers with irregular shapes, such as those in Kikka, Light Star or Veleta, displayed the lowest solidity values, but only Veleta had serrated flowers, while the asymmetry in Kikka and Light Star was caused by irregular petal arrangement within the flower. Other flower features with breeding interest that were not considered in this work are color and 3D shape. New image analysis software including some of these features is currently being implemented to overcome our current limitations in the description of flower morphologies and will be published elsewhere (Rolland-Lagan et al., in preparation).

Unlike carnation flowers whose morphology is rather simple, the petals have a complex morphology, with two distinct domains, the blade and the claw. For our morphometric analysis, we collected mature, fully-developed, petals from the outer whorl of the corolla. As for the flower area, petal area in standard carnation seemed to follow a bimodal distribution, suggesting a mixture of subpopulations of different sizes. In addition, petal convexity partly described the marginal serration of the petal blade, although this parameter was also influenced by the angle between the blade and the claw, hence for a similar level of marginal serration, obtuse angles increase their petal convexity values. For a better quantification of marginal serration in carnation petals, one should measure serration only in the more distal region of the petal where the serrations normally arise. Petal solidity accounted for the overall shape of the petal with the lowest values represented by petals with long and thin claws and small blades, such as those in Pink Amelie. To better define the spatial variation in the shape of the petals we used a program that systematically captures anchor points in the petal margin [12]. From the data generated by the analysis of 2200 petals, we identified eight principal components (PC) that gather about 90% of the observed variation in petal size and shape in cultivated carnation. Interestingly, the effect and the strength of the PCs identified were similar in standard (PC1 to PC4) and spray (pc1 to pc4) carnation cultivars, which is consistent with shared underlying mechanisms accounting for the morphological diversification of petals in both subpopulations. Alternatively, carnation breeders have systematically selected for similar traits in standard and spray carnation independently. Two of the PCs identified in petals captured most of the observed variation in the allometric size of the blade as well as the width of the claw. The third PC influences the bilateral symmetry of the petals and may be related to variations in petal phyllotaxis. Despite the subtle effect of the fourth PC in the total variation observed, this PC seems to specifically influence the shape of the blade. Still, this landmark-based method is unsuitable to capture the variation observed in the marginal serration of petals since tooth positioning and tooth size along the petal margin is not constantly spaced within the same cultivar, and the implementation of specific algorithms for tooth identification and quantification will allow us analyzing petal serration in detail.

We used a visualization method based on heat maps, which allowed us to establish similarity relationships between the different carnation cultivars studied. In our correlation analysis, we found that the size of the petals from the outer whorl contributed significantly to the size of the flower. Besides, petal number might also influence flower size and additional experiments with cultivars with similar petal size but distinct size of their flowers are ongoing in the laboratory to clarify this point. Although the dendrograms built with morphological characteristics might infer certain phylogenetic relationships of some cultivars, it is desirable to determine genetic relationships among the cultivated carnation cultivars using molecular markers. A new carnation map including 178 SSRs in 16 linkage groups covering 843.6 cM has been recently developed [17], and will facilitate the analysis of cultivated carnation phylogeny.

Since flowers were obtained from the same clonally-related mother plants of a given cultivar, variation found between replicates of the same cultivar might only be attributable to environmental causes, and to the age of the mother plant. We estimated the broad-sense heritabilities (*H^2^*) of some traits associated with size and shape of flowers and petals in a population of 78 carnation cultivars. In addition, by comparing replicates collected at different stages, we were able to identify a few cultivars whose phenotypes were strongly influenced by the environment, such as Kiro and Claudia, and it is anticipated that the establishment of regular flower production from this cultivars in different locations will be challenging. Additionally, we identified some cultivars with contrasting phenotypes which makes them candidate for the identification of the genes involved in the character under study through quantitative trait loci (QTL) analysis [18]. Indeed, since carnation cultivars are genetically heterogeneous [19], the F_1_ progeny between two established cultivars could be directly used, after genotyping the F_1_ seedlings with appropriate molecular markers, to map the genomic regions responsible for the observed phenotypic differences among F_1_ individuals.

In our experiment with carnation cultivars, flowers collected during autumn were smaller than those collected during winter, which suggested seasonal differences in petal growth. Alternatively, differences in the age of the mother plants might account for the growth differences observed between autumn (young mother plants) and winter (older mother plants). Our results from the time-course study of petal growth in the Master cultivar of standard carnation suggests that environmental differences between autumn and winter, rather than mother plant age, are responsible for the differential petal growth observed. Data on temperature, humidity and day-length duration from near weather stations are available (http://www.fomento.es/mfom/lang_castellano/). Flowers collected during the autumn developed from plants grown under long-day photoperiod and elevated temperatures in summer; while flowers collected during the winter developed from the same plants that were grown under short-days and lower temperatures in autumn. Additional studies are needed to determine the precise environmental cause(s) responsible for the observed variation in flower and petal size in carnation.

We previously reported a positive correlation between ploidy, cell size and petal size in cultivated carnation [20]. In our morphometric analysis, we identified several cultivars with contrasting petal size phenotypes that will allow the genetic dissection of endoreduplication thorough defined crosses and subsequent QTL analysis or by experimentally increasing their endoreduplication index. Our long-term goal is to provide carnation breeders with the essential molecular tools and markers to optimize their carnation breeding programs in order to develop new cultivars of higher value. Furthermore, our image analysis toolbox could easily be adapted to capture flower variation in other species with great ornamental value, such as cultivated rose and *Gerbera×hybrida*.

## Supporting Information

Figure S1 **Experimental design for flower phenotyping in cultivated carnation.** A, B, C and D represent clonally-related mother plants from the same cultivar. Ten mature flowers from different mother plants were harvested per cultivar at a given collection date. Four of these flowers (F1 to F4) were randomly chosen for flower morphometry and petal dissection. Five out of the seven petals taken from the outer whorl of these flowers were randomly chosen for petal morphometry (P1 to P20, shown in red). For those cultivars with two replicate, the second replicate was collected from the same mother plants and at a later time point; flowers and petals are numbered as F5 to F8 and P21 to P40, respectively.
(TIF)

Figure S2 **Other flower parameters measured.** (A, C) Histograms for the flower perimeter dataset with overlay of theoretical distributions (blue, normal distribution; red, log-normal distribution; pink, logistic distribution) in standard (A) and spray (C) cultivars. (B, D) Representative photographs of flowers with extreme values for perimeter in standard (B) and spray (D) cultivars. (E, G) Histograms for the flower AR dataset with overlay of theoretical distributions (blue, normal distribution; red, log-normal distribution; pink, logistic distribution) in standard (E) and spray (G) cultivars. (F, H) Representative photographs of flowers with extreme values for flower AR in standard (F) and spray (H) cultivars. Flower photographs were obtained and analyzed as described in Materials and methods.
(TIF)

Figure S3 **Other petal parameters measured.** (A, C) Histograms for the petal perimeter dataset with overlay of normal theoretical distribution in standard (A) and spray (C) cultivars. (B, D) Representative photographs of petals with extreme values for perimeter in standard (B) and spray (D) cultivars. (E, G) Histograms for the petal AR dataset with overlay of normal theoretical distribution in standard (E) and spray (G) cultivars. (F, H) Representative photographs of petals with extreme values for petal AR in standard (F) and spray (H) cultivars. Petal images were obtained and analyzed as described in Materials and methods.
(TIF)

Figure S4 **Three-dimensional PC map generated by standard deviations from the mean petal along PC1, PC2 and PC4 in standard carnation.** Each petal is represented as a point in a three dimensional space. The ellipses represent 1 SD from the mean petal for each one of the 55 nodes (cultivars) shown.
(TIF)

Figure S5 **Environmental-dependency of petal growth in** ***D. caryophyllus*** **.** Area and convexity values for flowers and petals are represented over collection date. Red, standard carnation data; green, spray carnation data. Linear regressions performed on data collected along the collecting season are also shown.
(TIF)

Table S1 **Carnation cultivars studied in this work.**
(DOCX)

Table S2 **Morphometric parameters studied in this work.**
(DOCX)

Table S3 **Statistical analysis of flower parameters.**
(DOCX)

Table S4 **Statistical analysis of petal parameters.**
(DOCX)

Table S5 **Linear correlation matrix for some flower and petal data.**
(DOCX)
