# Supplementary figures and images for: A Quantitative Framework for Flower Phenotyping in Cultivated Carnation (Dianthus caryophyllus L.)
Source: PLoS One. 2013 Dec 13;8(12):e82165. doi: 10.1371/journal.pone.0082165 (PMC3862579; doi:10.1371/journal.pone.0082165)

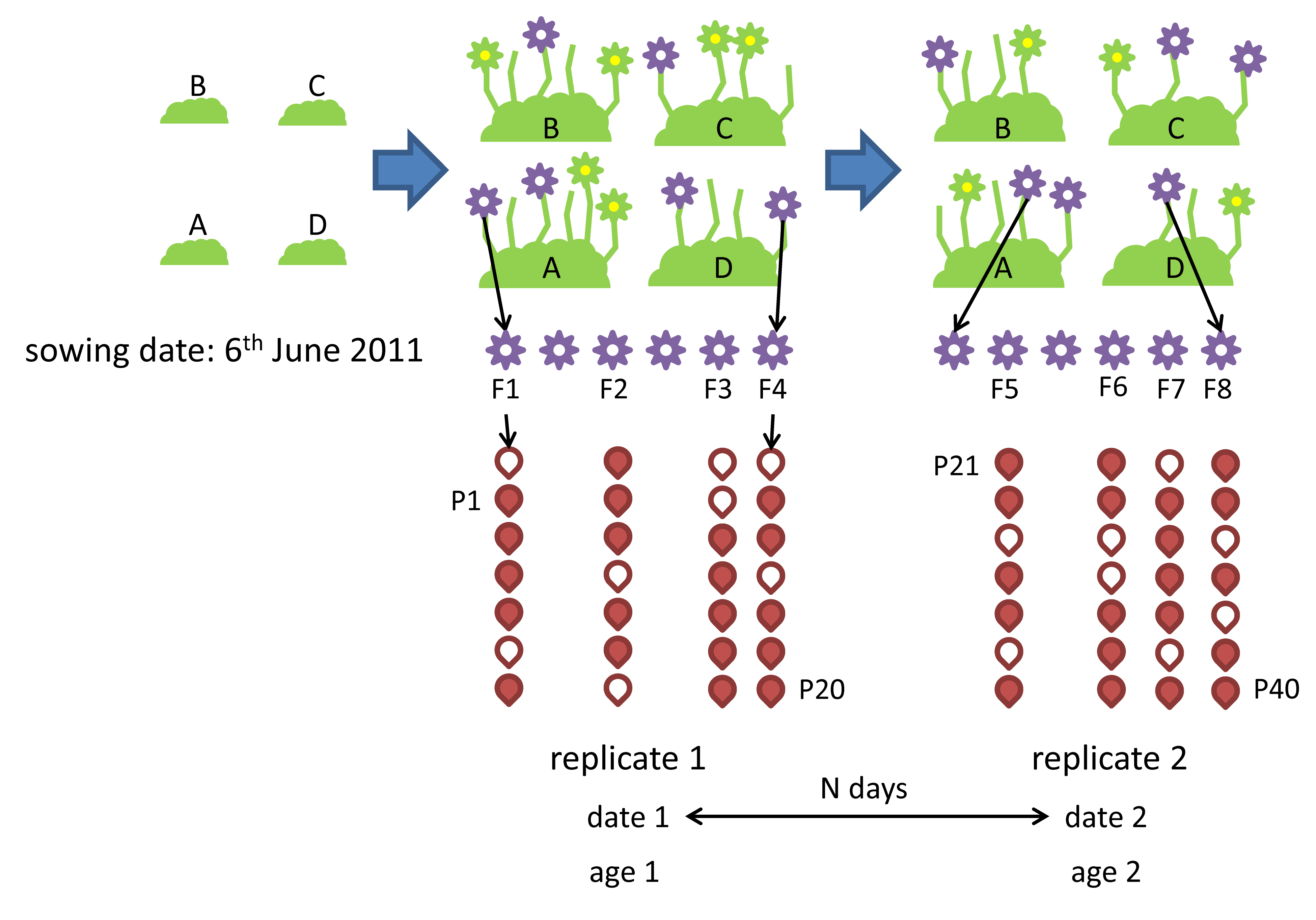

Supplement: Figure S1 — Experimental design for flower phenotyping in cultivated carnation. A, B, C and D represent clonally-related mother plants from the same cultivar. Ten mature flowers from different mother plants were harvested per cultivar at a given collection date. Four of these flowers (F1 to F4) were randomly chosen for flower morphometry and petal dissection. Five out of the seven petals taken from the outer whorl of these flowers were randomly chosen for petal morphometry (P1 to P20, shown in red). For those cultivars with two replicate, the second replicate was collected from the same mother plants and at a later time point; flowers and petals are numbered as F5 to F8 and P21 to P40, respectively. (TIF) [file pone.0082165.s001.tif]

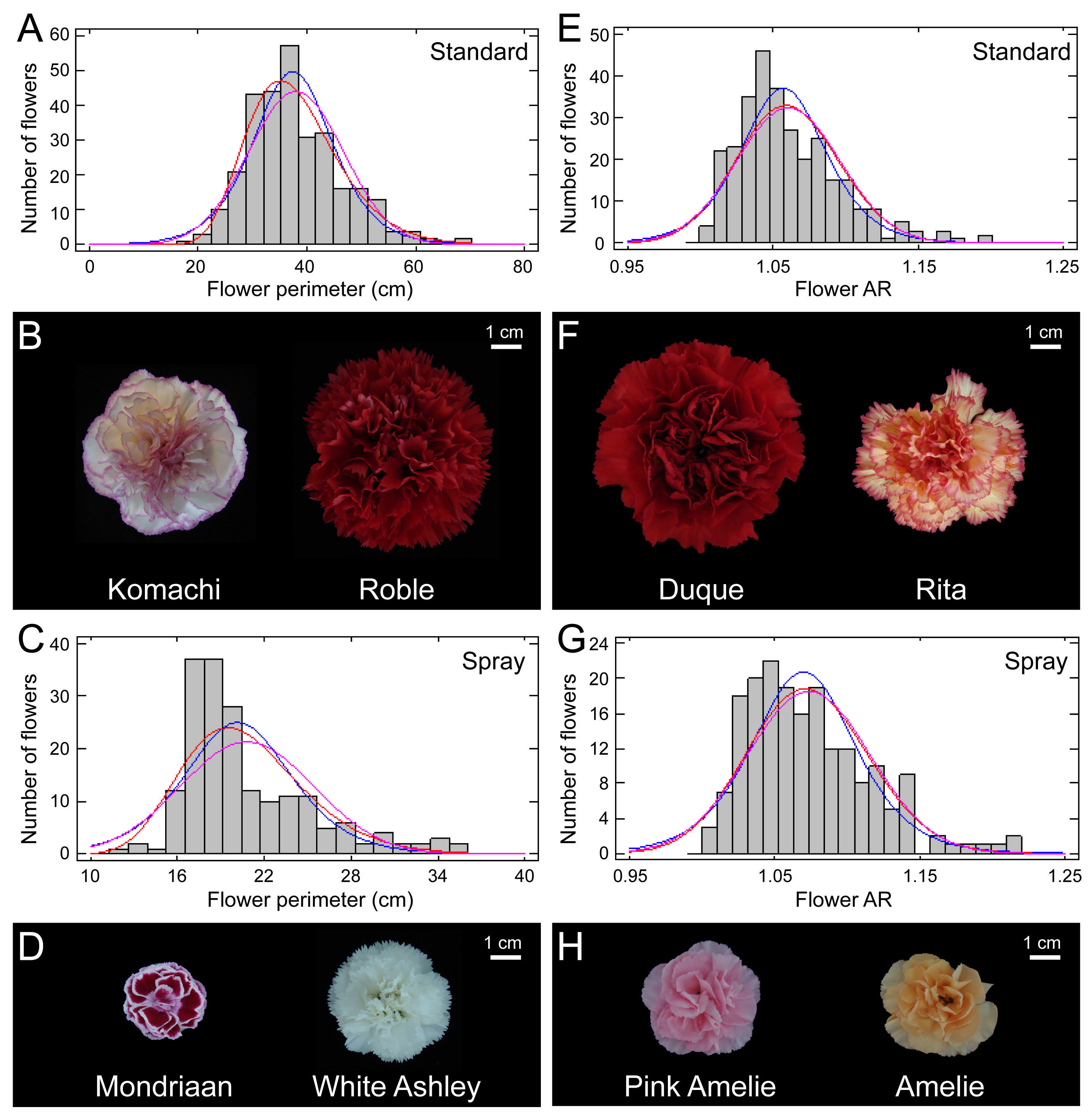

Supplement: Figure S2 — Other flower parameters measured. (A, C) Histograms for the flower perimeter dataset with overlay of theoretical distributions (blue, normal distribution; red, log-normal distribution; pink, logistic distribution) in standard (A) and spray (C) cultivars. (B, D) Representative photographs of flowers with extreme values for perimeter in standard (B) and spray (D) cultivars. (E, G) Histograms for the flower AR dataset with overlay of theoretical distributions (blue, normal distribution; red, log-normal distribution; pink, logistic distribution) in standard (E) and spray (G) cultivars. (F, H) Representative photographs of flowers with extreme values for flower AR in standard (F) and spray (H) cultivars. Flower photographs were obtained and analyzed as described in Materials and methods. (TIF) [file pone.0082165.s002.tif]

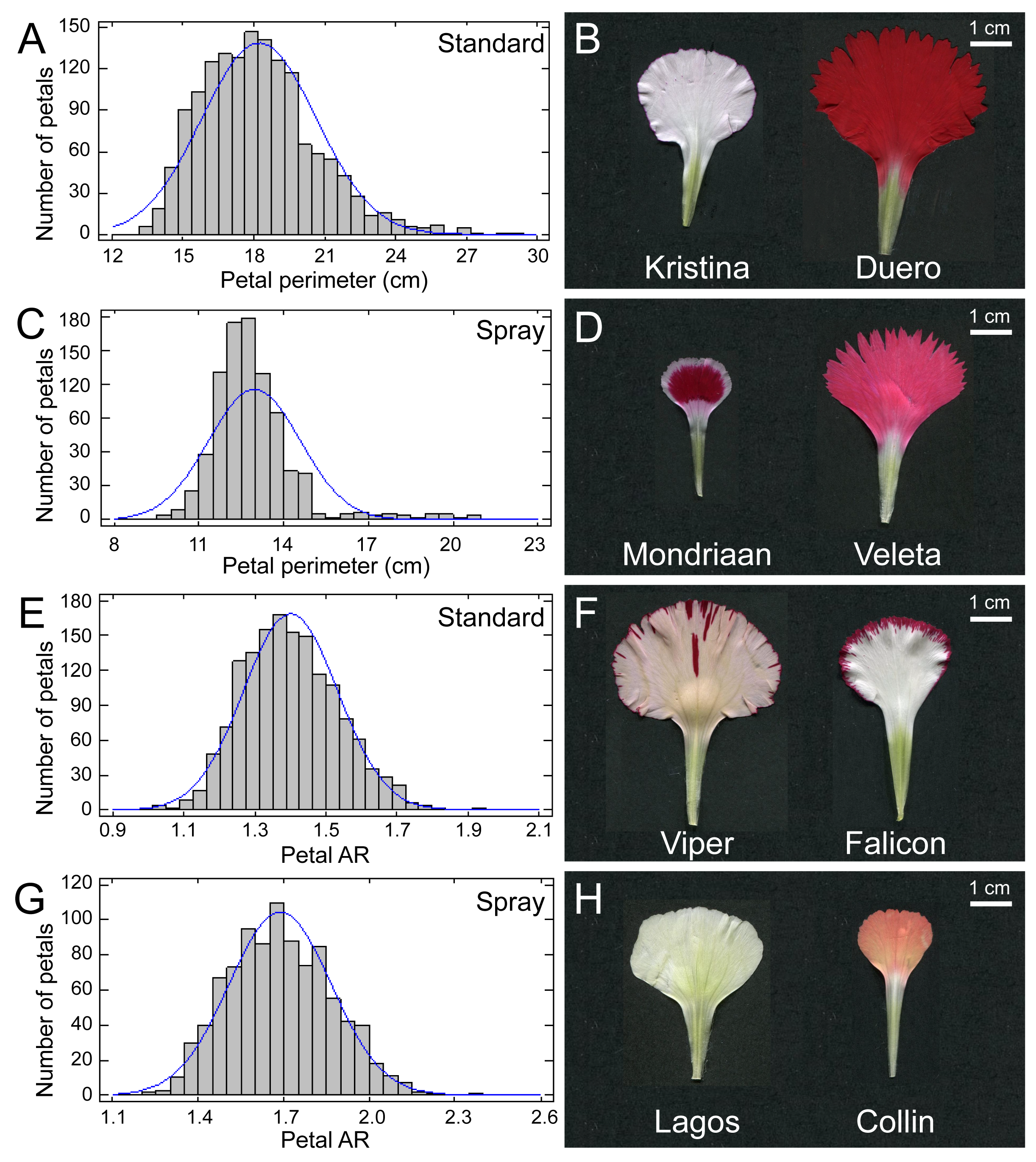

Supplement: Figure S3 — Other petal parameters measured. (A, C) Histograms for the petal perimeter dataset with overlay of normal theoretical distribution in standard (A) and spray (C) cultivars. (B, D) Representative photographs of petals with extreme values for perimeter in standard (B) and spray (D) cultivars. (E, G) Histograms for the petal AR dataset with overlay of normal theoretical distribution in standard (E) and spray (G) cultivars. (F, H) Representative photographs of petals with extreme values for petal AR in standard (F) and spray (H) cultivars. Petal images were obtained and analyzed as described in Materials and methods. (TIF) [file pone.0082165.s003.tif]

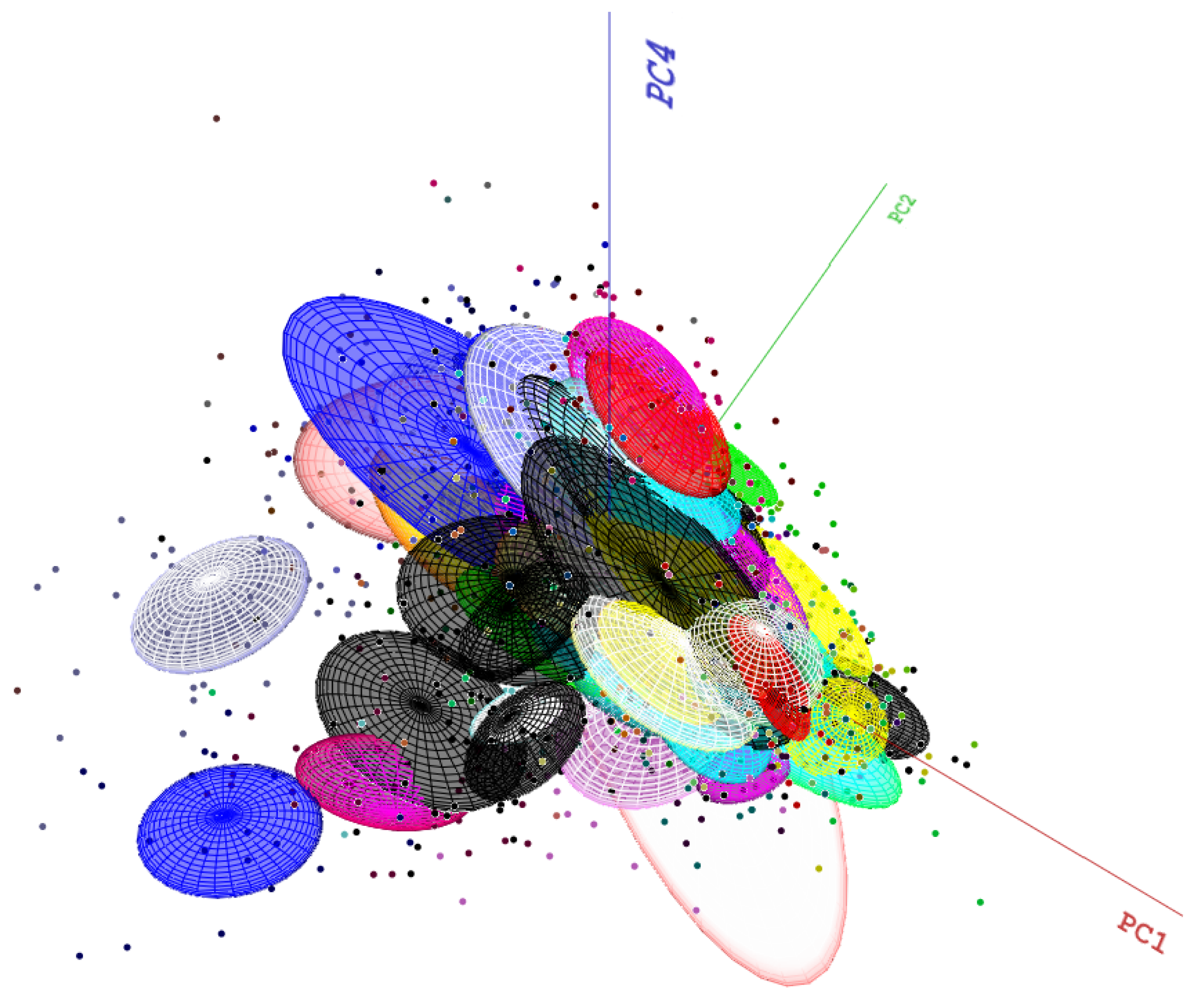

Supplement: Figure S4 — Three-dimensional PC map generated by standard deviations from the mean petal along PC1, PC2 and PC4 in standard carnation. Each petal is represented as a point in a three dimensional space. The ellipses represent 1 SD from the mean petal for each one of the 55 nodes (cultivars) shown. (TIF) [file pone.0082165.s004.tif]

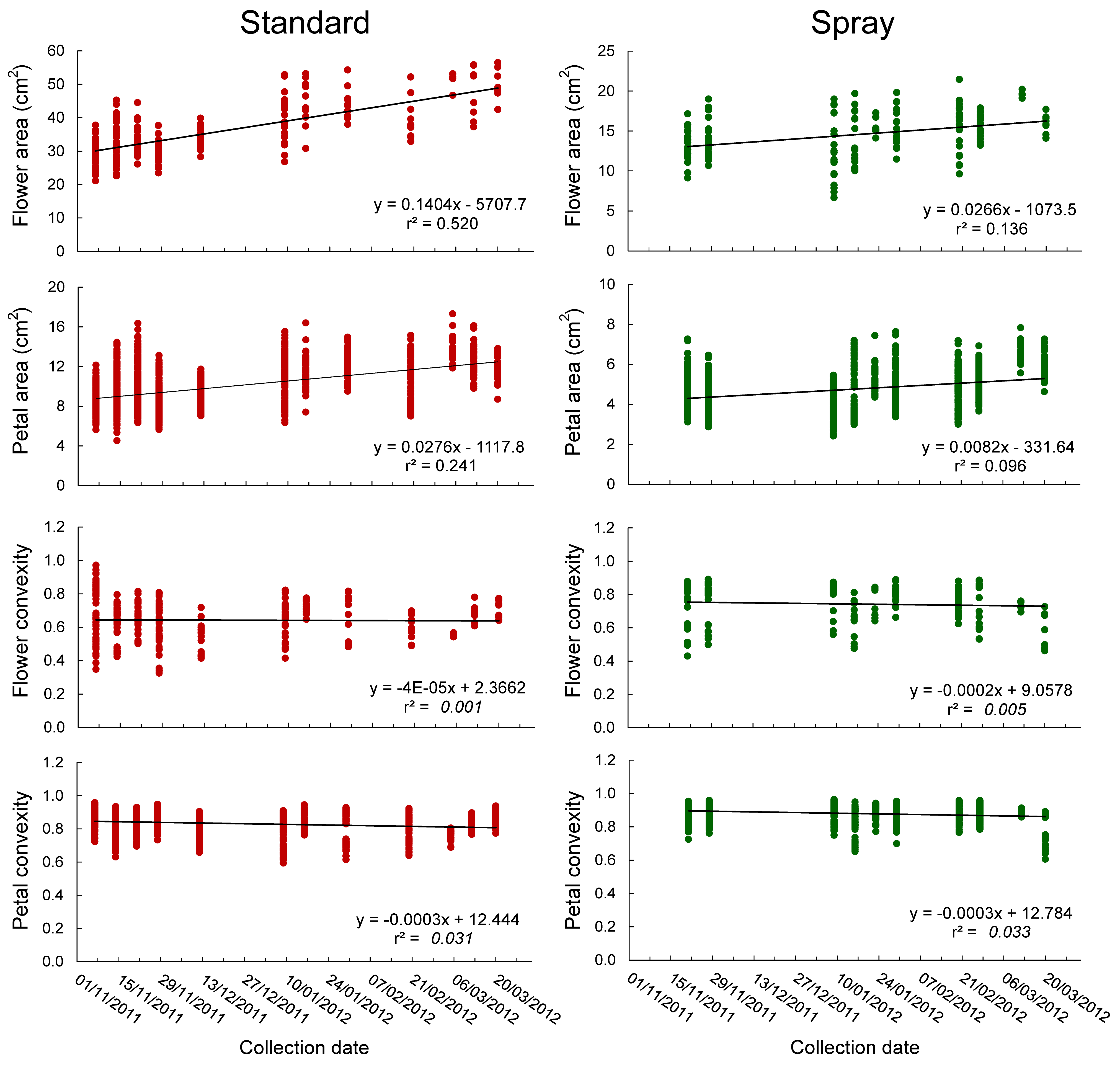

Supplement: Figure S5 — Environmental-dependency of petal growth in D. caryophyllus . Area and convexity values for flowers and petals are represented over collection date. Red, standard carnation data; green, spray carnation data. Linear regressions performed on data collected along the collecting season are also shown. (TIF) [file pone.0082165.s005.tif]
