# Supplementary material for: A Quantitative Framework for Flower Phenotyping in Cultivated Carnation (Dianthus caryophyllus L.)
Source: PLoS One. 2013 Dec 13;8(12):e82165. doi: 10.1371/journal.pone.0082165 (PMC3862579; doi:10.1371/journal.pone.0082165)
Supplement: Table S1 — Carnation cultivars studied in this work. (DOCX) [file pone.0082165.s006.docx]

**Table S1.- Carnation cultivars studied in this work**

| Commercial name | Cultivar type | Owner |
| --- | --- | --- |
| Algar | Standard | Barberet & Blanc |
| Alicia | Standard | Barberet & Blanc |
| Apple Tea | Standard | Moraglia |
| Arte | Standard | Barberet & Blanc |
| Atenea | Standard | Barberet & Blanc |
| Benidorm | Standard | Barberet & Blanc |
| Benji | Standard | Barberet & Blanc |
| Black Baccara | Standard | Barberet & Blanc |
| Borja | Standard | Barberet & Blanc |
| Casper | Standard | La Villetta |
| Ceres | Standard | Moraglia |
| Clara | Standard | Barberet & Blanc |
| Coralie | Standard | Barberet & Blanc |
| Delicia | Standard | Barberet & Blanc |
| Domingo | Standard | Barberet & Blanc |
| Duero | Standard | Barberet & Blanc |
| Dumas | Standard | Barberet & Blanc |
| Duque | Standard | Barberet & Blanc |
| Falicon | Standard | Barberet & Blanc |
| Famosa | Standard | Barberet & Blanc |
| Fiesta Komachi | Standard | Barberet & Blanc |
| Franky | Standard | Barberet & Blanc |
| Fuente | Standard | Barberet & Blanc |
| Holly | Standard | Barberet & Blanc |
| Hugo | Standard | Barberet & Blanc |
| Inka | Standard | Barberet & Blanc |
| Kafka | Standard | Barberet & Blanc |
| Kikka | Standard | Barberet & Blanc |
| Kiro | Standard | Barberet & Blanc |
| Komachi | Standard | Barberet & Blanc |
| Komachi Blanco | Standard | Barberet & Blanc |
| Kristina | Standard | Barberet & Blanc |
| Light Star | Standard | Barberet & Blanc |
| Lorca | Standard | Barberet & Blanc |
| Madame Augier | Standard | Barberet & Blanc |
| Marielle | Standard | Barberet & Blanc |
| Master | Standard | Barberet & Blanc |
| Megu | Standard | Barberet & Blanc |
| Mojacar | Standard | Barberet & Blanc |
| Paola | Standard | Barberet & Blanc |
| Paris | Standard | Barberet & Blanc |
| Pilar | Standard | Barberet & Blanc |
| Pink Dover | Standard | Barberet & Blanc |
| Purias | Standard | Barberet & Blanc |
| Reina | Standard | Barberet & Blanc |
| Reina Nieve | Standard | Barberet & Blanc |
| Rita | Standard | Barberet & Blanc |
| Roble | Standard | Barberet & Blanc |
| Rosalba | Standard | Barberet & Blanc |
| Snap | Standard | Barberet & Blanc |
| Star | Standard | Barberet & Blanc |
| Star Fire | Standard | Barberet & Blanc |
| Vinko | Standard | Barberet & Blanc |
| Viper | Standard | Selecta Klemm |
| Viper Wine | Standard | Selecta Klemm |
| Amelie | Spray | Hirugami |
| Arcos | Spray | Barberet & Blanc |
| Aveiro | Spray | Barberet & Blanc |
| Cerise Amelie | Spray | Hirugami |
| Claudia | Spray | Barberet & Blanc |
| Collin | Spray | Hirugami |
| Collin Lemon | Spray | Hirugami |
| Galaxia | Spray | Barberet & Blanc |
| Guadalupe | Spray | West Select, B.V. |
| Lagos | Spray | Barberet & Blanc |
| Light Cream Candle | Spray | Inagaki |
| Luxor | Spray | Barberet & Blanc |
| Milky Way | Spray | Barberet & Blanc |
| Montana | Spray | Barberet & Blanc |
| Pink Amelie | Spray | Hirugami |
| Pino Rosso | Spray | Di Giorgio |
| Promesa | Spray | Barberet & Blanc |
| Rocio | Spray | Barberet & Blanc |
| Rose Candle | Spray | Inagaki |
| Veleta | Spray | Barberet & Blanc |
| White Ashley | Spray | Barberet & Blanc |
| Wish | Spray | Barberet & Blanc |
| Mondriaan | Pot | Barberet & Blanc |
