# Supplementary material for: A Quantitative Framework for Flower Phenotyping in Cultivated Carnation (Dianthus caryophyllus L.)
Source: PLoS One. 2013 Dec 13;8(12):e82165. doi: 10.1371/journal.pone.0082165 (PMC3862579; doi:10.1371/journal.pone.0082165)
Supplement: Table S2 — Morphometric parameters studied in this work. (DOCX) [file pone.0082165.s007.docx]

**Table S2.- Morphometric parameters studied in this work**

| Name | Symbol | Calculated as | Unit |
| --- | --- | --- | --- |
| Area | A | - | cm^2^ |
| Perimeter | P | - | cm |
| Ellipse major chord length | EM | - | cm |
| Ellipse minor chord length | EN | - | cm |
| Convex hull area | HA | - | cm^2^ |
| Convex hull perimeter | HP | - | cm |
| Aspect ratio | AR | EM/EN | - |
| Solidity | S | A/HA | - |
| Convexity | C | HP/P | - |
| Estimated volume | EV | 2/3π(EM/2)^3^ | cm^3^ |
