# Supplementary material for: A Quantitative Framework for Flower Phenotyping in Cultivated Carnation (Dianthus caryophyllus L.)
Source: PLoS One. 2013 Dec 13;8(12):e82165. doi: 10.1371/journal.pone.0082165 (PMC3862579; doi:10.1371/journal.pone.0082165)
Supplement: Table S3 — Statistical analysis of flower parameters. (DOCX) [file pone.0082165.s008.docx]

**Table S3.- Statistical analysis of flower parameters**

3A.- Linear correlation matrix

|  | A (top) | P (top) | AR (top) | S (top) | C (top) | A (side) | P (side) | AR (side) | S (side) | C (side) | EV |
| --- | --- | --- | --- | --- | --- | --- | --- | --- | --- | --- | --- |
| A (top) |  | 0.426 | -0.147 | *-0.077* | *-0.016* | **0.752** | 0.569 | *0.053* | *-0.095* | *-0.065* | **0.985** |
| P (top) | 0.423 |  | *-0.066* | -0.527 | **-0.878** | 0.234 | 0.464 | 0.214 | -0.137 | -0.421 | 0.415 |
| AR (top) | -0.228 | *-0.070* |  | -0.149 | *0.003* | *-0.037* | -0.147 | *-0.098* | *0.081* | 0.150 | *0.004* |
| S (top) | *0.038* | -0.662 | -0.265 |  | 0.581 | *-0.092* | -0.227 | -0.152 | *0.106* | 0.248 | *-0.104* |
| C (top) | *0.003* | **-0.885** | *-0.011* | **0.736** |  | *0.059* | -0.279 | -0.199 | 0.129 | 0.459 | *-0.012* |
| A (side) | **0.824** | 0.334 | *-0.130* | *-0.001* | *0.004* |  | 0.623 | -0.503 | 0.182 | *-0.069* | **0.751** |
| P (side) | 0.633 | 0.587 | *-0.104* | -0.267 | -0.352 | 0.655 |  | -0.218 | -0.375 | **-0.782** | 0.547 |
| AR (side) | *-0.134* | *-0.051* | *-0.047* | *0.007* | *-0.006* | -0.505 | -0.332 |  | -0.315 | *0.051* | *0.038* |
| S (side) | *0.084* | *-0.011* | *0.008* | *0.089* | *0.044* | *0.130* | -0.326 | *0.028* |  | 0.470 | *-0.086* |
| C (side) | *-0.041* | -0.472 | *0.021* | 0.366 | 0.505 | *-0.018* | **-0.726** | *0.126* | 0.442 |  | *-0.042* |
| EV | **0.976** | 0.421 | *-0.028* | *-0.018* | *-0.006* | **0.818** | 0.623 | *-0.132* | *0.088* | *-0.037* |  |

Non-significant correlations (*P* > 0.05) for flower data are shown in italics. Top half: standard carnation (n=268), bottom half: spray and pot carnation (n=172). R^2^ values higher than 50% are shown in bold.

3B.- Descriptive statistics

| Cultivar | N |  | Area (cm^2^) | |  | Solidity | |  | Convexity | |
| --- | --- | --- | --- | --- | --- | --- | --- | --- | --- | --- |
|  |  |  | Mean±SD | m, M |  | Mean±SD | m, M |  | Mean±SD | m, M |
| Algar | 4 |  | 35.95±1.81 | 34.31, 37.89 |  | 0.923±0.015 | 0.906, 0.942 |  | 0.657±0.049 | 0.594, 0.698 |
| Alicia | 4 |  | 28.41±1.45 | 26.88, 30.37 |  | 0.932±0.003 | 0.930, 0.937 |  | 0.480±0.041 | 0.432, 0.532 |
| Apple Tea | 4 |  | 29.91±2.36 | 27.93, 32.91 |  | 0.926±0.028 | 0.884, 0.947 |  | 0.731±0.022 | 0.703, 0.755 |
| Arte | 4 |  | 29.80±1.58 | 28.28, 32.02 |  | 0.931±0.015 | 0.912, 0.948 |  | 0.575±0.033 | 0.537, 0.614 |
| Atenea | 4 |  | 42.56±2.53 | 39.60, 45.26 |  | 0.939±0.005 | 0.933, 0.946 |  | 0.726±0.017 | 0.708, 0.747 |
| Benidorm | 4 |  | 35.03±2.43 | 32.21, 37.72 |  | 0.969±0.012 | 0.954, 0.980 |  | 0.831±0.050 | 0.768, 0.887 |
| Benji | 4 |  | 49.03±4.52 | 43.23, 52.84 |  | 0.920±0.008 | 0.911, 0.928 |  | 0.680±0.062 | 0.608, 0.739 |
| Black Baccara | 4 |  | 25.46±4.53 | 21.08, 31.81 |  | 0.968±0.010 | 0.954, 0.977 |  | 0.939±0.024 | 0.919, 0.971 |
| Borja | 4 |  | 34.28±4.42 | 30.85, 40.24 |  | 0.943±0.011 | 0.933, 0.957 |  | 0.634±0.048 | 0.573, 0.675 |
| Casper | 4 |  | 33.95±2.15 | 32.29, 37.10 |  | 0.913±0.014 | 0.898, 0.932 |  | 0.515±0.059 | 0.431, 0.564 |
| Ceres | 4 |  | 29.52±1.85 | 27.70, 31.93 |  | 0.948±0.009 | 0.939, 0.958 |  | 0.694±0.035 | 0.658, 0.741 |
| Clara | 8 |  | 42.14±8.22 | 26.83, 54.25 |  | 0.926±0.031 | 0.861, 0.954 |  | 0.690±0.048 | 0.617, 0.761 |
| Coralie | 4 |  | 38.23±1.71 | 36.76, 39.97 |  | 0.958±0.009 | 0.944, 0.963 |  | 0.719±0.037 | 0.671, 0.752 |
| Delicia | 4 |  | 39.02±3.93 | 35.93, 44.49 |  | 0.925±0.012 | 0.911, 0.941 |  | 0.670±0.055 | 0.616, 0.745 |
| Domingo | 4 |  | 30.83±4.22 | 26.13, 34.40 |  | 0.914±0.011 | 0.901, 0.924 |  | 0.508±0.008 | 0.501, 0.519 |
| Duero | 8 |  | 45.71±3.99 | 39.60, 52.14 |  | 0.923±0.012 | 0.907, 0.945 |  | 0.511±0.033 | 0.482, 0.578 |
| Dumas | 4 |  | 35.27±2.84 | 32.07, 38.81 |  | 0.895±0.018 | 0.869, 0.912 |  | 0.452±0.008 | 0.440, 0.459 |
| Duque | 4 |  | 40.51±3.22 | 37.24, 44.48 |  | 0.915±0.025 | 0.884, 0.944 |  | 0.719±0.043 | 0.681, 0.779 |
| Falicon | 8 |  | 26.85±3.13 | 22.56, 31.21 |  | 0.921±0.019 | 0.885, 0.943 |  | 0.543±0.129 | 0.348, 0.682 |
| Famosa | 4 |  | 32.42±0.60 | 31.59, 33.03 |  | 0.922±0.023 | 0.893, 0.948 |  | 0.551±0.021 | 0.538, 0.582 |
| Fiesta Komachi | 4 |  | 28.44±1.08 | 26.89, 29.38 |  | 0.938±0.011 | 0.928, 0.954 |  | 0.778±0.041 | 0.719, 0.808 |
| Franky | 4 |  | 28.76±3.16 | 25.41, 32.89 |  | 0.965±0.004 | 0.960, 0.969 |  | 0.845±0.055 | 0.802, 0.924 |
| Fuente | 4 |  | 30.90±2.07 | 28.02, 32.79 |  | 0.946±0.008 | 0.935, 0.953 |  | 0.647±0.030 | 0.616, 0.676 |
| Holly | 8 |  | 44.95±7.06 | 34.27, 53.09 |  | 0.905±0.014 | 0.882, 0.923 |  | 0.608±0.063 | 0.542, 0.691 |
| Hugo | 4 |  | 33.21±3.67 | 28.08, 36.23 |  | 0.936±0.015 | 0.918, 0.953 |  | 0.696±0.071 | 0.655, 0.802 |
| Inka | 8 |  | 32.04±5.69 | 26.11, 39.84 |  | 0.945±0.010 | 0.932, 0.965 |  | 0.684±0.064 | 0.596, 0.795 |
| Kafka | 8 |  | 51.49±3.37 | 46.47, 56.44 |  | 0.928±0.017 | 0.895, 0.950 |  | 0.741±0.036 | 0.671, 0.775 |
| Kikka | 8 |  | 28.96±2.73 | 25.59, 32.49 |  | 0.885±0.024 | 0.859, 0.927 |  | 0.525±0.040 | 0.465, 0.578 |
| Kiro | 8 |  | 32.89±2.73 | 29.39, 37.74 |  | 0.917±0.016 | 0.891, 0.943 |  | 0.656±0.054 | 0.570, 0.715 |
| Komachi | 8 |  | 29.19±1.76 | 26.75, 32.26 |  | 0.966±0.009 | 0.948, 0.976 |  | 0.844±0.027 | 0.796, 0.876 |
| Komachi Blanco | 4 |  | 30.11±3.50 | 25.16, 33.39 |  | 0.954±0.002 | 0.952, 0.956 |  | 0.664±0.038 | 0.624, 0.701 |
| Kristina | 4 |  | 24.20±1.63 | 22.75, 26.49 |  | 0.944±0.006 | 0.937, 0.950 |  | 0.752±0.050 | 0.684, 0.791 |
| Light Star | 4 |  | 31.63±2.72 | 29.03, 35.22 |  | 0.886±0.034 | 0.837, 0.910 |  | 0.616±0.038 | 0.559, 0.639 |
| Lorca | 4 |  | 30.82±0.40 | 30.35, 31.25 |  | 0.927±0.014 | 0.911, 0.944 |  | 0.640±0.012 | 0.628, 0.652 |
| Madame Augier | 4 |  | 33.47±1.63 | 32.06, 35.81 |  | 0.904±0.011 | 0.897, 0.920 |  | 0.684±0.031 | 0.654, 0.722 |
| Marielle | 4 |  | 34.63±2.73 | 31.85, 37.13 |  | 0.903±0.030 | 0.860, 0.929 |  | 0.655±0.044 | 0.619, 0.719 |
| Master | 8 |  | 34.86±3.77 | 30.86, 42.70 |  | 0.901±0.004 | 0.893, 0.908 |  | 0.448±0.031 | 0.414, 0.498 |
| Megu | 4 |  | 44.97±0.27 | 44.73, 45.35 |  | 0.956±0.003 | 0.953, 0.959 |  | 0.783±0.041 | 0.728, 0.821 |
| Mojacar | 4 |  | 31.01±1.42 | 29.84, 33.07 |  | 0.916±0.020 | 0.897, 0.938 |  | 0.468±0.066 | 0.387, 0.530 |
| Paola | 4 |  | 27.10±3.45 | 23.46, 30.47 |  | 0.903±0.025 | 0.871, 0.931 |  | 0.539±0.012 | 0.523, 0.551 |
| Paris | 4 |  | 32.80±0.35 | 32.56, 33.31 |  | 0.942±0.005 | 0.938, 0.949 |  | 0.784±0.028 | 0.756, 0.814 |
| Pilar | 4 |  | 32.95±2.88 | 30.60, 36.54 |  | 0.934±0.008 | 0.923, 0.942 |  | 0.714±0.028 | 0.674, 0.741 |
| Pink Dover | 4 |  | 31.86±3.15 | 28.32, 35.33 |  | 0.924±0.011 | 0.911, 0.936 |  | 0.569±0.017 | 0.546, 0.586 |
| Purias | 4 |  | 32.12±4.16 | 28.10, 37.65 |  | 0.916±0.010 | 0.907, 0.930 |  | 0.404±0.128 | 0.326, 0.596 |
| Reina | 4 |  | 35.92±3.22 | 32.42, 39.94 |  | 0.924±0.012 | 0.913, 0.936 |  | 0.673±0.044 | 0.614, 0.716 |
| Reina Nieve | 4 |  | 34.61±5.76 | 29.59, 39.69 |  | 0.917±0.015 | 0.904, 0.930 |  | 0.549±0.023 | 0.514, 0.563 |
| Rita | 4 |  | 26.83±1.32 | 25.60, 28.05 |  | 0.897±0.025 | 0.866, 0.927 |  | 0.677±0.028 | 0.636, 0.694 |
| Roble | 4 |  | 37.72±2.92 | 34.13, 41.08 |  | 0.913±0.010 | 0.903, 0.922 |  | 0.457±0.026 | 0.424, 0.482 |
| Rosalba | 4 |  | 39.65±1.15 | 37.97, 40.54 |  | 0.947±0.004 | 0.943, 0.951 |  | 0.794±0.023 | 0.768, 0.815 |
| Snap | 4 |  | 31.50±1.84 | 29.06, 33.02 |  | 0.921±0.011 | 0.906, 0.932 |  | 0.528±0.055 | 0.484, 0.607 |
| Star | 4 |  | 29.64±4.41 | 26.13, 36.08 |  | 0.880±0.010 | 0.872, 0.893 |  | 0.622±0.048 | 0.571, 0.681 |
| Star Fire | 4 |  | 31.72±3.25 | 28.16, 34.69 |  | 0.874±0.016 | 0.859, 0.894 |  | 0.602±0.040 | 0.568, 0.651 |
| Vinko | 4 |  | 28.78±0.35 | 28.37, 29.07 |  | 0.897±0.026 | 0.871, 0.920 |  | 0.643±0.047 | 0.586, 0.684 |
| Viper | 8 |  | 49.13±6.98 | 37.47, 55.81 |  | 0.913±0.014 | 0.883, 0.930 |  | 0.655±0.028 | 0.608, 0.691 |
| Viper Wine | 8 |  | 42.78±5.76 | 30.76, 48.35 |  | 0.919±0.010 | 0.908, 0.932 |  | 0.704±0.050 | 0.640, 0.764 |
| Amelie | 8 |  | 14.55±2.19 | 12.60, 17.79 |  | 0.931±0.012 | 0.905, 0.941 |  | 0.823±0.026 | 0.790, 0.857 |
| Arcos | 4 |  | 10.67±0.88 | 10.01, 11.96 |  | 0.912±0.012 | 0.896, 0.926 |  | 0.658±0.011 | 0.648, 0.669 |
| Aveiro | 8 |  | 10.12±1.27 | 7.36, 11.20 |  | 0.914±0.021 | 0.867, 0.933 |  | 0.656±0.060 | 0.559, 0.729 |
| Cerise Amelie | 4 |  | 14.26±1.48 | 12.17, 15.51 |  | 0.940±0.021 | 0.912, 0.964 |  | 0.572±0.195 | 0.429, 0.861 |
| Claudia | 8 |  | 13.40±3.12 | 9.12, 17.83 |  | 0.910±0.014 | 0.892, 0.931 |  | 0.583±0.033 | 0.531, 0.624 |
| Collin | 4 |  | 11.98±1.16 | 10.68, 13.51 |  | 0.933±0.017 | 0.916, 0.958 |  | 0.816±0.020 | 0.797, 0.837 |
| Collin Lemon | 8 |  | 13.62±1.58 | 11.34, 15.95 |  | 0.945±0.009 | 0.934, 0.957 |  | 0.837±0.035 | 0.770, 0.880 |
| Galaxia | 8 |  | 18.50±1.69 | 16.17, 21.45 |  | 0.914±0.013 | 0.892, 0.928 |  | 0.707±0.046 | 0.624, 0.770 |
| Guadalupe | 12 |  | 12.47±0.70 | 11.41, 13.75 |  | 0.917±0.007 | 0.909, 0.934 |  | 0.797±0.032 | 0.724, 0.835 |
| Lagos | 8 |  | 17.72±0.89 | 16.34, 18.99 |  | 0.938±0.018 | 0.910, 0.968 |  | 0.820±0.038 | 0.746, 0.88 |
| Light Cream Candle | 8 |  | 15.57±1.40 | 13.55, 17.38 |  | 0.959±0.009 | 0.947, 0.971 |  | 0.824±0.047 | 0.761, 0.886 |
| Luxor | 8 |  | 12.82±1.22 | 11.15, 14.13 |  | 0.936±0.008 | 0.924, 0.950 |  | 0.777±0.026 | 0.747, 0.812 |
| Milky Way | 8 |  | 18.94±1.02 | 17.48, 20.21 |  | 0.917±0.025 | 0.883, 0.952 |  | 0.702±0.041 | 0.641, 0.760 |
| Montana | 8 |  | 15.45±2.70 | 11.97, 18.99 |  | 0.944±0.010 | 0.929, 0.961 |  | 0.829±0.026 | 0.796, 0.874 |
| Pink Amelie | 8 |  | 13.95±1.79 | 11.55, 17.15 |  | 0.955±0.007 | 0.943, 0.963 |  | 0.856±0.021 | 0.817, 0.891 |
| Pino Rosso | 8 |  | 16.04±1.29 | 14.11, 17.71 |  | 0.903±0.024 | 0.876, 0.935 |  | 0.670±0.043 | 0.588, 0.727 |
| Promesa | 8 |  | 14.83±0.53 | 13.78, 15.35 |  | 0.949±0.015 | 0.921, 0.966 |  | 0.847±0.025 | 0.819, 0.889 |
| Rocio | 8 |  | 14.64±1.32 | 12.95, 17.02 |  | 0.900±0.017 | 0.872, 0.918 |  | 0.620±0.046 | 0.551, 0.698 |
| Rose Candle | 8 |  | 13.93±2.25 | 11.06, 17.87 |  | 0.949±0.022 | 0.903, 0.970 |  | 0.783±0.118 | 0.512, 0.856 |
| Veleta | 8 |  | 15.42±1.42 | 12.62, 16.74 |  | 0.875±0.010 | 0.862, 0.892 |  | 0.490±0.027 | 0.462, 0.546 |
| White Ashley | 4 |  | 14.22±1.11 | 12.80, 15.25 |  | 0.922±0.014 | 0.903, 0.937 |  | 0.535±0.032 | 0.498, 0.576 |
| Wish | 12 |  | 16.50±1.54 | 13.28, 18.66 |  | 0.954±0.010 | 0.935, 0.965 |  | 0.822±0.029 | 0.761, 0.852 |
| Mondriaan | 4 |  | 8.09±1.24 | 6.64, 9.65 |  | 0.956±0.003 | 0.954, 0.962 |  | 0.854±0.018 | 0.831, 0.873 |
