# Supplementary material for: A Quantitative Framework for Flower Phenotyping in Cultivated Carnation (Dianthus caryophyllus L.)
Source: PLoS One. 2013 Dec 13;8(12):e82165. doi: 10.1371/journal.pone.0082165 (PMC3862579; doi:10.1371/journal.pone.0082165)
Supplement: Table S4 — Statistical analysis of petal parameters. (DOCX) [file pone.0082165.s009.docx]

**Table S4.- Statistical analysis of petal parameters**

4A.- Linear correlation matrix

|  | Area | Perimeter | AR | Solidity | Convexity |
| --- | --- | --- | --- | --- | --- |
| Area |  | **0.745** | **-0.782** | 0.533 | -0.280 |
| Perimeter | **0.691** |  | -0.541 | 0.021 | **-0.801** |
| AR | **-0.737** | -0.498 |  | -0.540 | 0.303 |
| Solidity | 0.478 | -0.092 | -0.486 |  | 0.232 |
| Convexity | -0.142 | **-0.728** | 0.328 | 0.284 |  |

All correlations were highly significant (*P* < 0.005). Top half: standard carnation (n=1340), bottom half: spray and pot carnation (n=860). R^2^ values higher than 50% are shown in bold.

4B.- Descriptive statistics

| Cultivar | N |  | Area (cm^2^) | |  | Solidity | |  | Convexity | |
| --- | --- | --- | --- | --- | --- | --- | --- | --- | --- | --- |
|  |  |  | Mean±SD | m, M |  | Mean±SD | m, M |  | Mean±SD | m, M |
| Algar | 20 |  | 7.87±0.85 | 6.98, 11.01 |  | 0.774±0.018 | 0.738, 0.808 |  | 0.874±0.028 | 0.838, 0.923 |
| Alicia | 20 |  | 9.13±1.34 | 7.13, 11.73 |  | 0.758±0.016 | 0.731, 0.783 |  | 0.842±0.030 | 0.792, 0.889 |
| Apple Tea | 20 |  | 8.81±1.28 | 6.86, 11.38 |  | 0.773±0.014 | 0.755, 0.807 |  | 0.903±0.032 | 0.830, 0.948 |
| Arte | 20 |  | 8.30±0.41 | 7.42, 8.94 |  | 0.755±0.022 | 0.724, 0.802 |  | 0.795±0.031 | 0.723, 0.832 |
| Atenea | 20 |  | 12.25±3.05 | 8.29, 19.87 |  | 0.769±0.034 | 0.704, 0.829 |  | 0.809±0.052 | 0.660, 0.890 |
| Benidorm | 20 |  | 10.01±0.71 | 8.98, 11.61 |  | 0.783±0.018 | 0.755, 0.821 |  | 0.896±0.036 | 0.793, 0.938 |
| Benji | 20 |  | 13.46±1.04 | 11.68, 15.52 |  | 0.824±0.019 | 0.786, 0.861 |  | 0.824±0.031 | 0.735, 0.875 |
| Black Baccara | 20 |  | 7.21±0.64 | 5.61, 8.17 |  | 0.766±0.025 | 0.715, 0.817 |  | 0.893±0.028 | 0.841, 0.952 |
| Borja | 20 |  | 10.41±1.49 | 7.90, 13.43 |  | 0.774±0.016 | 0.734, 0.803 |  | 0.798±0.043 | 0.691, 0.849 |
| Casper | 20 |  | 8.91±0.78 | 7.24, 10.33 |  | 0.695±0.024 | 0.646, 0.736 |  | 0.790±0.030 | 0.741, 0.854 |
| Ceres | 20 |  | 7.86±0.48 | 6.84, 8.97 |  | 0.748±0.010 | 0.721, 0.763 |  | 0.884±0.025 | 0.834, 0.927 |
| Clara | 40 |  | 12.21±1.30 | 9.50, 14.96 |  | 0.808±0.021 | 0.735, 0.836 |  | 0.865±0.022 | 0.816, 0.909 |
| Coralie | 20 |  | 12.05±1.62 | 9.73, 14.43 |  | 0.797±0.012 | 0.781, 0.823 |  | 0.834±0.026 | 0.784, 0.878 |
| Delicia | 20 |  | 10.76±1.51 | 8.87, 15.73 |  | 0.790±0.017 | 0.752, 0.826 |  | 0.854±0.039 | 0.770, 0.915 |
| Domingo | 20 |  | 7.86±0.82 | 6.56, 9.54 |  | 0.733±0.016 | 0.703, 0.755 |  | 0.802±0.031 | 0.757, 0.861 |
| Duero | 40 |  | 12.76±1.06 | 10.75, 15.14 |  | 0.765±0.016 | 0.728, 0.801 |  | 0.693±0.035 | 0.614, 0.750 |
| Dumas | 20 |  | 8.90±0.93 | 7.50, 10.84 |  | 0.760±0.017 | 0.735, 0.792 |  | 0.697±0.034 | 0.630, 0.775 |
| Duque | 20 |  | 12.29±1.54 | 9.80, 16.10 |  | 0.825±0.015 | 0.797, 0.861 |  | 0.856±0.025 | 0.801, 0.896 |
| Falicon | 40 |  | 7.90±1.06 | 5.78, 9.80 |  | 0.748±0.019 | 0.689, 0.799 |  | 0.870±0.025 | 0.815, 0.914 |
| Famosa | 20 |  | 6.61±0.40 | 5.65, 7.24 |  | 0.741±0.020 | 0.699, 0.777 |  | 0.856±0.018 | 0.808, 0.886 |
| Fiesta Komachi | 20 |  | 9.20±1.10 | 6.77, 11.45 |  | 0.760±0.014 | 0.727, 0.783 |  | 0.887±0.027 | 0.830, 0.936 |
| Franky | 20 |  | 9.33±0.85 | 7.83, 10.79 |  | 0.796±0.020 | 0.754, 0.836 |  | 0.889±0.042 | 0.815, 0.957 |
| Fuente | 20 |  | 9.07±0.65 | 7.90, 10.11 |  | 0.789±0.019 | 0.761, 0.836 |  | 0.817±0.027 | 0.779, 0.876 |
| Holly | 40 |  | 12.92±1.65 | 10.13, 17.30 |  | 0.767±0.019 | 0.726, 0.801 |  | 0.790±0.044 | 0.688, 0.866 |
| Hugo | 20 |  | 8.02±0.78 | 6.48, 9.03 |  | 0.779±0.016 | 0.752, 0.817 |  | 0.870±0.031 | 0.791, 0.921 |
| Inka | 40 |  | 9.62±1.60 | 7.28, 13.71 |  | 0.783±0.019 | 0.744, 0.814 |  | 0.860±0.037 | 0.795, 0.924 |
| Kafka | 40 |  | 12.00±1.15 | 9.64, 13.81 |  | 0.792±0.018 | 0.746, 0.821 |  | 0.896±0.027 | 0.828, 0.944 |
| Kikka | 40 |  | 7.30±0.82 | 6.00, 9.12 |  | 0.746±0.022 | 0.711, 0.796 |  | 0.836±0.040 | 0.706, 0.910 |
| Kiro | 40 |  | 9.03±1.29 | 7.07, 13.53 |  | 0.789±0.015 | 0.746, 0.819 |  | 0.871±0.048 | 0.774, 0.933 |
| Komachi | 40 |  | 8.79±1.30 | 6.29, 11.32 |  | 0.746±0.016 | 0.712, 0.775 |  | 0.894±0.024 | 0.843, 0.945 |
| Komachi Blanco | 20 |  | 9.45±2.04 | 5.79, 13.12 |  | 0.770±0.013 | 0.746, 0.793 |  | 0.861±0.029 | 0.810, 0.904 |
| Kristina | 20 |  | 7.74±1.03 | 6.14, 9.51 |  | 0.789±0.016 | 0.767, 0.824 |  | 0.894±0.018 | 0.859, 0.917 |
| Light Star | 20 |  | 8.75±1.24 | 6.66, 11.59 |  | 0.751±0.018 | 0.724, 0.777 |  | 0.856±0.030 | 0.801, 0.902 |
| Lorca | 20 |  | 8.00±1.25 | 6.55, 11.02 |  | 0.782±0.027 | 0.739, 0.833 |  | 0.878±0.026 | 0.811, 0.924 |
| Madame Augier | 20 |  | 11.78±2.31 | 7.88, 16.35 |  | 0.761±0.031 | 0.697, 0.833 |  | 0.786±0.041 | 0.695, 0.860 |
| Marielle | 20 |  | 10.53±2.14 | 7.75, 15.05 |  | 0.768±0.027 | 0.722, 0.805 |  | 0.817±0.042 | 0.738, 0.895 |
| Master | 40 |  | 8.05±0.69 | 7.02, 9.90 |  | 0.705±0.019 | 0.664, 0.742 |  | 0.689±0.042 | 0.593, 0.755 |
| Megu | 20 |  | 12.69±1.12 | 10.2, 14.22 |  | 0.821±0.015 | 0.797, 0.846 |  | 0.846±0.025 | 0.802, 0.893 |
| Mojacar | 20 |  | 8.78±1.13 | 6.78, 10.48 |  | 0.770±0.042 | 0.704, 0.882 |  | 0.868±0.033 | 0.783, 0.911 |
| Paola | 20 |  | 7.91±0.62 | 6.99, 9.16 |  | 0.772±0.017 | 0.742, 0.804 |  | 0.791±0.032 | 0.732, 0.847 |
| Paris | 20 |  | 10.20±1.29 | 8.40, 12.52 |  | 0.814±0.022 | 0.774, 0.855 |  | 0.898±0.016 | 0.866, 0.929 |
| Pilar | 20 |  | 10.01±1.45 | 7.74, 12.93 |  | 0.808±0.015 | 0.784, 0.838 |  | 0.837±0.043 | 0.764, 0.916 |
| Pink Dover | 20 |  | 9.41±1.26 | 7.17, 11.48 |  | 0.752±0.022 | 0.722, 0.785 |  | 0.810±0.025 | 0.769, 0.850 |
| Purias | 20 |  | 10.30±1.62 | 7.43, 12.62 |  | 0.763±0.021 | 0.714, 0.794 |  | 0.831±0.026 | 0.776, 0.877 |
| Reina | 20 |  | 10.65±2.55 | 7.19, 14.27 |  | 0.773±0.026 | 0.728, 0.808 |  | 0.804±0.038 | 0.722, 0.858 |
| Reina Nieve | 20 |  | 9.42±2.71 | 6.37, 14.93 |  | 0.774±0.023 | 0.725, 0.811 |  | 0.832±0.050 | 0.746, 0.900 |
| Rita | 20 |  | 10.09±1.81 | 7.01, 12.81 |  | 0.741±0.018 | 0.704, 0.768 |  | 0.829±0.044 | 0.734, 0.895 |
| Roble | 20 |  | 8.89±1.48 | 6.93, 13.44 |  | 0.717±0.022 | 0.681, 0.752 |  | 0.728±0.033 | 0.668, 0.780 |
| Rosalba | 20 |  | 11.17±0.88 | 9.85, 13.28 |  | 0.795±0.018 | 0.754, 0.821 |  | 0.889±0.024 | 0.832, 0.928 |
| Snap | 20 |  | 9.07±1.25 | 7.49, 12.14 |  | 0.764±0.027 | 0.719, 0.813 |  | 0.862±0.035 | 0.812, 0.920 |
| Star | 20 |  | 7.29±1.45 | 4.51, 10.20 |  | 0.716±0.022 | 0.656, 0.756 |  | 0.868±0.031 | 0.810, 0.913 |
| Star Fire | 20 |  | 7.64±1.10 | 6.12, 10.35 |  | 0.710±0.016 | 0.676, 0.732 |  | 0.890±0.028 | 0.818, 0.934 |
| Vinko | 20 |  | 9.21±2.10 | 6.32, 12.47 |  | 0.763±0.029 | 0.704, 0.808 |  | 0.817±0.039 | 0.761, 0.883 |
| Viper | 40 |  | 11.95±1.64 | 9.41, 16.39 |  | 0.790±0.022 | 0.745, 0.830 |  | 0.819±0.028 | 0.766, 0.862 |
| Viper Wine | 40 |  | 11.14±1.27 | 7.39, 14.69 |  | 0.788±0.020 | 0.744, 0.840 |  | 0.823±0.025 | 0.762, 0.865 |
| Amelie | 40 |  | 4.66±0.75 | 3.28, 6.22 |  | 0.736±0.036 | 0.677, 0.809 |  | 0.920±0.026 | 0.834, 0.953 |
| Arcos | 20 |  | 3.65±0.39 | 3.13, 4.37 |  | 0.703±0.019 | 0.676, 0.754 |  | 0.822±0.022 | 0.784, 0.862 |
| Aveiro | 40 |  | 3.33±0.32 | 2.42, 3.92 |  | 0.723±0.021 | 0.683, 0.761 |  | 0.823±0.026 | 0.749, 0.864 |
| Cerise Amelie | 20 |  | 4.76±0.73 | 3.38, 5.95 |  | 0.713±0.024 | 0.665, 0.750 |  | 0.909±0.021 | 0.851, 0.931 |
| Claudia | 40 |  | 4.37±0.68 | 3.33, 5.77 |  | 0.728±0.023 | 0.688, 0.769 |  | 0.807±0.027 | 0.724, 0.847 |
| Collin | 20 |  | 4.00±0.71 | 2.87, 5.22 |  | 0.700±0.020 | 0.667, 0.729 |  | 0.929±0.017 | 0.889, 0.947 |
| Collin Lemon | 40 |  | 5.11±0.55 | 3.59, 6.33 |  | 0.761±0.032 | 0.714, 0.823 |  | 0.930±0.016 | 0.895, 0.959 |
| Galaxia | 40 |  | 5.64±0.87 | 4.01, 7.64 |  | 0.735±0.022 | 0.672, 0.780 |  | 0.838±0.038 | 0.699, 0.886 |
| Guadalupe | 60 |  | 3.90±0.44 | 3.07, 5.76 |  | 0.709±0.025 | 0.658, 0.768 |  | 0.895±0.033 | 0.772, 0.950 |
| Lagos | 40 |  | 4.84±0.76 | 3.80, 6.60 |  | 0.775±0.029 | 0.709, 0.822 |  | 0.935±0.018 | 0.897, 0.964 |
| Light Cream Candle | 40 |  | 5.82±0.63 | 4.49, 7.27 |  | 0.746±0.033 | 0.646, 0.795 |  | 0.903±0.025 | 0.836, 0.956 |
| Luxor | 40 |  | 3.77±0.44 | 2.98, 4.53 |  | 0.732±0.019 | 0.686, 0.769 |  | 0.909±0.018 | 0.861, 0.949 |
| Milky Way | 40 |  | 6.43±0.57 | 5.52, 7.83 |  | 0.784±0.022 | 0.737, 0.818 |  | 0.887±0.020 | 0.839, 0.919 |
| Montana | 40 |  | 4.64±0.67 | 3.55, 5.95 |  | 0.724±0.023 | 0.665, 0.762 |  | 0.923±0.015 | 0.876, 0.951 |
| Pink Amelie | 40 |  | 4.60±0.59 | 3.36, 5.78 |  | 0.703±0.025 | 0.628, 0.754 |  | 0.916±0.022 | 0.865, 0.948 |
| Pino Rosso | 40 |  | 5.73±0.46 | 5.09, 6.89 |  | 0.732±0.018 | 0.666, 0.766 |  | 0.862±0.026 | 0.772, 0.891 |
| Promesa | 40 |  | 4.98±0.66 | 4.29, 7.44 |  | 0.709±0.017 | 0.677, 0.751 |  | 0.922±0.016 | 0.889, 0.952 |
| Rocio | 40 |  | 4.44±0.58 | 3.56, 6.46 |  | 0.739±0.031 | 0.678, 0.814 |  | 0.850±0.031 | 0.795, 0.919 |
| Rose Candle | 40 |  | 4.72±0.66 | 3.12, 5.99 |  | 0.742±0.025 | 0.693, 0.795 |  | 0.908±0.016 | 0.869, 0.943 |
| Veleta | 40 |  | 6.08±0.73 | 4.63, 7.26 |  | 0.699±0.020 | 0.647, 0.737 |  | 0.687±0.033 | 0.605, 0.752 |
| White Ashley | 20 |  | 3.94±0.38 | 3.21, 4.59 |  | 0.682±0.014 | 0.657, 0.706 |  | 0.824±0.033 | 0.760, 0.865 |
| Wish | 60 |  | 5.07±0.58 | 4.02, 6.92 |  | 0.769±0.017 | 0.715, 0.808 |  | 0.918±0.020 | 0.859, 0.948 |
| Mondriaan | 20 |  | 3.08±0.46 | 2.41, 4.22 |  | 0.708±0.019 | 0.667, 0.742 |  | 0.892±0.019 | 0.864, 0.926 |
