# Supplementary material for: A Quantitative Framework for Flower Phenotyping in Cultivated Carnation (Dianthus caryophyllus L.)
Source: PLoS One. 2013 Dec 13;8(12):e82165. doi: 10.1371/journal.pone.0082165 (PMC3862579; doi:10.1371/journal.pone.0082165)
Supplement: Table S5 — Linear correlation matrix for some flower and petal data. (DOCX) [file pone.0082165.s010.docx]

**Table S5.- Linear correlation matrix for some flower and petal data**

|  | A (flower. top) | AR (flower. top) | S (flower. top) | C (flower. top) | A (petal) | AR (petal) | S (petal) | C (petal) |
| --- | --- | --- | --- | --- | --- | --- | --- | --- |
| A (flower. top) |  | -0.147 | *-0.077* | *-0.016* | **0.794** | -0.547 | 0.373 | -0.297 |
| AR (flower. top) | -0.228 |  | -0.149 | *0.003* | *-0.035* | *0.080* | *-0.044* | 0.154 |
| S (flower. top) | *0.038* | -0.265 |  | 0.581 | *0.016* | *-0.107* | 0.327 | 0.367 |
| C (flower. top) | *0.003* | *-0.011* | 0.736 |  | 0.137 | *-0.064* | 0.452 | 0.595 |
| A (petal) | **0.789** | -0.146 | *-0.088* | *-0.094* |  | **-0.782** | 0.549 | -0.255 |
| AR (petal) | -0.517 | 0.219 | 0.170 | 0.259 | **-0.720** |  | -0.593 | 0.284 |
| S (petal) | 0.533 | -0.238 | 0.179 | 0.239 | 0.455 | -0.502 |  | 0.290 |
| C (petal) | *0.065* | *-0.047* | **0.693** | **0.790** | *-0.132* | 0.366 | 0.319 |  |

Non-significant correlations (*P* > 0.005) are shown in italics. Top half: standard carnation, bottom half: spray and pot carnation. R^2^ values higher than 50% are shown in bold.
